# Massive histone H3 phosphorylation without mitotic chromosome condensation

**DOI:** 10.1016/j.isci.2026.117718

**Published:** 2026-09-22

**Authors:** Ipek Ustun, Marco Borsò, Natalia Y. Kochanova, Ignasi Forne, Caitlin Reid, Bram Prevo, Kumiko Samejima, Linfeng Xie, James R. Paulson, Axel Imhof, William C. Earnshaw

**Affiliations:** 1 Institute for Cell Biology, University of Edinburgh, Swann Building, King’s Buildings, Max Born Crescent, Edinburgh EH9 3B, UK; 2 Protein Analysis Unit, Biomedical Center (BMC), LMU Munich, Planegg-Martinsried, Germany; 3 Department of Chemistry, University of Wisconsin-Oshkosh, Oshkosh, WI, USA

**Keywords:** Biological sciences, Molecular biology, Chromosome organization

## Abstract

Histone post-translational modifications change dramatically as cells enter mitosis. Indeed, histone phosphorylation and deacetylation have been proposed to be major factors driving the compaction of mitotic chromosomes. However, whether these modification changes directly regulate mitotic chromatin compaction remains a subject of debate. Here, we use a CDK1^as^ chemical-genetic system to accumulate cells in G_2_ phase. Treatment of these G_2_ cells with the phosphatase inhibitor calyculin A efficiently causes them to undergo premature chromosome condensation, even when the CDK1^as^ allele is inhibited with 1NM-PP1. Surprisingly, simultaneous treatment of the cells with R547, an inhibitor of CDK1, 2, and 4 kinases, causes them to remain phenotypically in G_2_ phase. Strikingly, although mitotic-like chromatin condensation fails to occur in these cells, LC-MS reveals that histone H3 phosphorylation occurs even more efficiently than in a normal mitosis. Furthermore, histone deacetylation also occurs in those interphase-looking calyculin A-treated cells. These results reveal that histone phosphorylation and deacetylation are not sufficient to drive mitotic-like chromosome condensation and compaction.

## Highlights

• Calyculin A induces PCC in DT40 cells arrested in G2 phase by CDK1 inhibition
• R547 or Palbociclib inhibition suggests CDK2/CDK4 as secondary drivers of PCC
• Calyculin A induces massive H3S10ph and H3T3ph even when PCC is prevented
• Calyculin-A induces histone deacetylation even when PCC is prevented

## Graphical abstract

**Figure:**
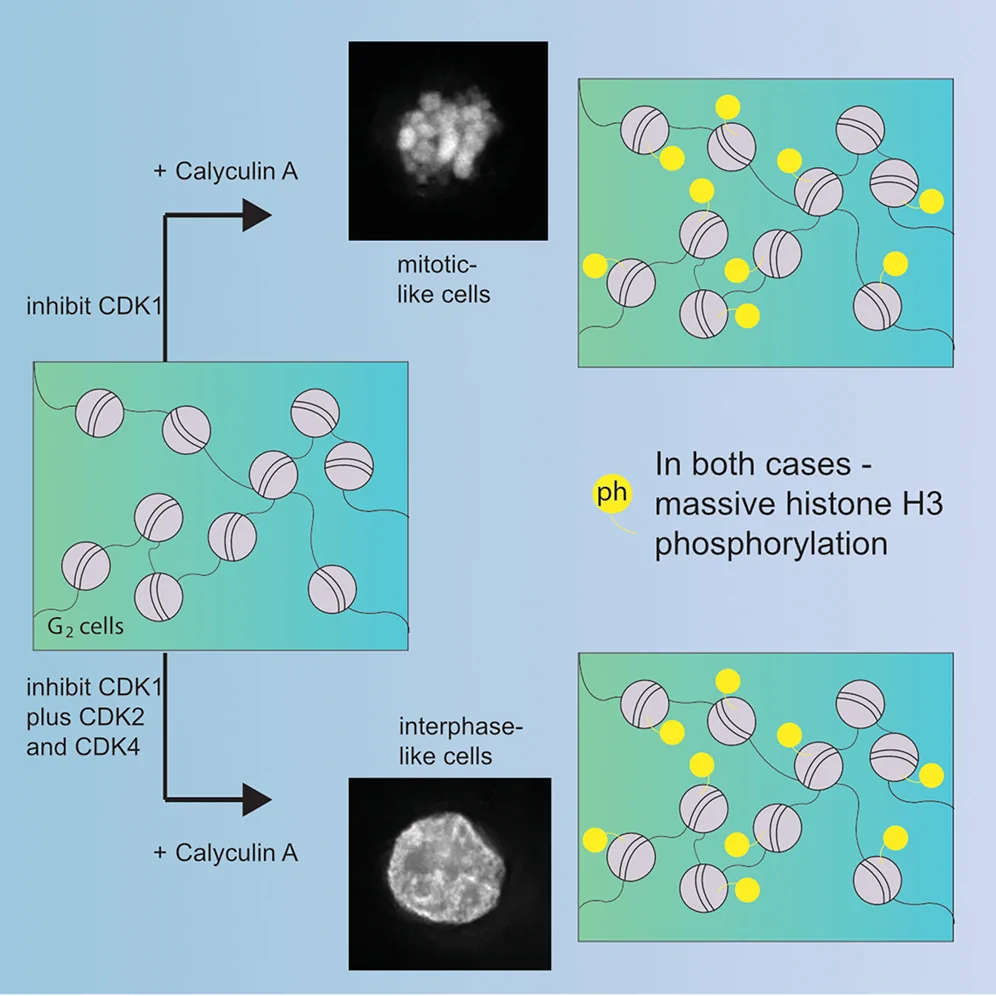

## Introduction

The timely condensation of DNA into x-shaped chromosomes in mitosis is one of the most evocative structural transitions in cell biology. This process is accompanied by genome-wide changes in histone phosphorylation and acetylation. Mitotic histone phosphorylation is carried out by the serine/threonine kinases aurora B and haspin. The corresponding deacetylation is functionally less studied but is at least partially carried out by HDAC3.^1^ These changes have been proposed to directly contribute to chromosome condensation,^2^^,^^3^^,^^4^^,^^5^^,^^6^ but to date this hypothesis has had limited experimental confirmation in cells.

Mitotic structural transitions are triggered by cyclin-dependent kinases (CDKs). CDK1 activated primarily by cyclin B triggers the transition from G_2_ phase into mitosis.^7^^,^^8^^,^^9^^,^^10^^,^^11^ CDK2 drives the G_1_/S and S/G_2_ transitions through interactions with cyclins E and A.^11^^,^^12^ CDK4/6 bound to cyclin D regulate cell cycle progression at the restriction point in part via phosphorylation of the retinoblastoma protein.^11^^,^^12^ Recently, it was shown that CDK5 can also form a complex with cyclin B that safeguards mitotic fidelity.^13^

CDK1 has classically been believed to be necessary and sufficient for cell division.^14^^,^^15^ Indeed, in a chemical-genetic system, inhibition of a CDK1^as^ allele with 1NM-PP1 is sufficient to block cells in G_2_.^16^^,^^17^^,^^18^ However, recent studies revealed that CDK2 overexpression in a CDK1-deficient background can restore the ability of cells to divide. In this context, CDK2 functions in a complex with cyclin B.^19^ Furthermore, cyclin A overexpression can rescue the phenotype of cyclin B deficiency.^20^ These experiments highlight partial redundancies between CDKs1/2 and cyclins A/B. Additional plasticity in cell cycle regulation is revealed by the observation that CDK1 can also drive the G_1_-S transition by binding cyclin D in the absence of CDK4/6^14^^,^^21^ and cyclin E in the absence of CDK2.^19^^,^^22^

It is now widely believed that the access of a wide range of factors to chromatin is regulated by post-translational modifications (PTMs) of the core histones that serve as epigenetic marks.^23^^,^^24^^,^^25^^,^^26^^,^^27^ These PTMs are regulated throughout the cell cycle in dividing cells^5^^,^^28^^,^^29^^,^^30^^,^^31^^,^^32^^,^^33^^,^^34^^,^^35^ and help to set the transcriptional profile of differentiated cells.^36^^,^^37^ Hallmarks of mitosis include phosphorylation of histone H3 on threonine 3,^38^ serine 10^39^^,^^40^ and serine 28^41^ (H3T3ph, H3S10ph, and H3S28ph, respectively), as well as serine 31 of histone H3.3 (H3.3S31ph),^42^ and threonine 120 of histone H2A (H2AT120ph).^43^

H3T3 phosphorylation by haspin kinase helps to recruit the chromosomal passenger complex to centromeres.^44^^,^^45^^,^^46^^,^^47^ More recently, H3T3ph was found to be a general mitosis-specific modification of heterochromatin during mitotic entry.^28^ The role of this genome-wide modification of T3 remains unexplored. Likewise, the role of H3S10 phosphorylation (by aurora B kinase in the chromosomal passenger complex - CPC)^48^^,^^49^^,^^50^ has been unclear. A role for H3S10ph in chromosome condensation was originally suggested based on genetic studies in *Tetrahymena*^51^ and budding yeast.^4^^,^^6^ However, in mammalian cells, rod-shaped chromosomes form following chemical inhibition of aurora B^52^ and injection of antibody to H3S10ph leads to chromosome misalignment, but rod-shaped compact mitotic chromosomes still form.^53^

Mitotic chromosome condensation is regulated by a balance of kinase and phosphatase activities. Indeed, okadaic acid and calyculin A (inhibitors of the serine/threonine phosphatases PP1 and PP2A)^54^^,^^55^^,^^56^ can directly induce premature chromosome condensation (PCC) in interphase mammalian cells. These prematurely condensed chromosomes resemble mitotic chromosomes and phenocopy the classic PCC phenotype induced by fusion of interphase and mitotic cells.^57^

Here, we used a CDK1^as^ system to accumulate cultures of chicken DT40 cells in G_2_ phase. However, instead of washing out the 1NM-PP1 inhibitor and releasing the cells into mitosis as in previous studies,^18^^,^^28^^,^^58^^,^^59^ we treated cells with calyculin A to induce PCC. This system allowed us to functionally separate chromosome condensation from segregation.

Calyculin A-treated cells undergo dramatic chromosome condensation. The fact that these cells fail to divide within 4 h suggests that the process corresponds to PCC rather than a premature entry into conventional mitosis. CDK1 inhibition by 1NM-PP1 did not block this PCC; however, supplementing the 1NM-PP1 with R547 (inhibitor of CDK1, CDK2, and CDK4) prevented the condensation pathway and cells remained morphologically in G_2_. Together, these inhibitor treatments suggested that calyculin-induced PCC is mainly driven by contributions from CDK1 and/or CDK2 (and possibly CDK4). Strikingly, liquid chromatography-mass spectroscopy (LC-MS) of isolated histones revealed that, in G_2_-arrested cells with decondensed chromatin in the presence of calyculin A plus R547, histone Hyper-phosphorylation and deacetylation showed levels similar to or exceeding those in condensed mitotic chromosomes. Our results suggest that CDK2 at physiological levels can contribute to mitotic chromosome condensation and furthermore indicate that extensive histone phosphorylation and deacetylation can be uncoupled from chromosome condensation.

## Results

### Calyculin A causes premature chromosome condensation in CDK1^as^-blocked cells

To examine the role of different CDKs in driving mitotic chromosome condensation, we used a chemical-genetic system based on a CDK1^as^ cell line for uniform synchronization of chicken DT40 cells. Treatment of these cells with the ATP analog 1NM-PP1 blocks CDK1 activation and efficiently arrests the cell population in G_2_.^17^^,^^18^ To determine the ability of chromosomes in these cells to undergo mitotic-like condensation, we treated cultures with calyculin A, an inhibitor of protein phosphatases 1 and 2A, that has previously been shown to efficiently induce PCC in cultured cells.^55^

To test the effect of calyculin A on chromosome condensation in the absence of CDK1 activity, we treated G_2_-arrested cells in presence of 1NM-PP1 with 100 nM calyculin A for 7.5, 15, 30, and 60 min and followed the progression of PCC over time (Figure 1A). Hoechst 33342 staining and immunofluorescence staining for lamin B1 revealed the progress of PCC at different time-points (Figures 1B and 1C). We classified cells into four different phenotypes depending on the state of chromosome condensation as judged by DNA staining and nuclear envelope breakdown as judged by the lamin B1 staining. PCC was not yet visible at 7.5 min of calyculin A treatment (Figures 1C; Table S1). The first noticeable condensation was visible at 15 min near the nuclear rim (“rim” phenotype). At this early stage, the nuclear envelope appeared fully intact as in mitotic prophase.^18^ By 30 min 8 ± 0.4% of the cells exhibited more condensed chromatin with breaks in the nuclear envelope (“mixed” phenotype). By 60 min 80% of the cells had lost their nuclear envelope. Of these cells, 28 ± 13% exhibited condensed chromosomes that were scattered inside the cell (“scattered” phenotype) while in 53 ± 6% of cells, the chromatin was clustered with no space between chromosomes (“compact” phenotype). We focused our subsequent investigations on the 30- and 60-min timepoints as they were reminiscent of the prophase and prometaphase stages of conventional mitotic entry.

**Figure 1:**
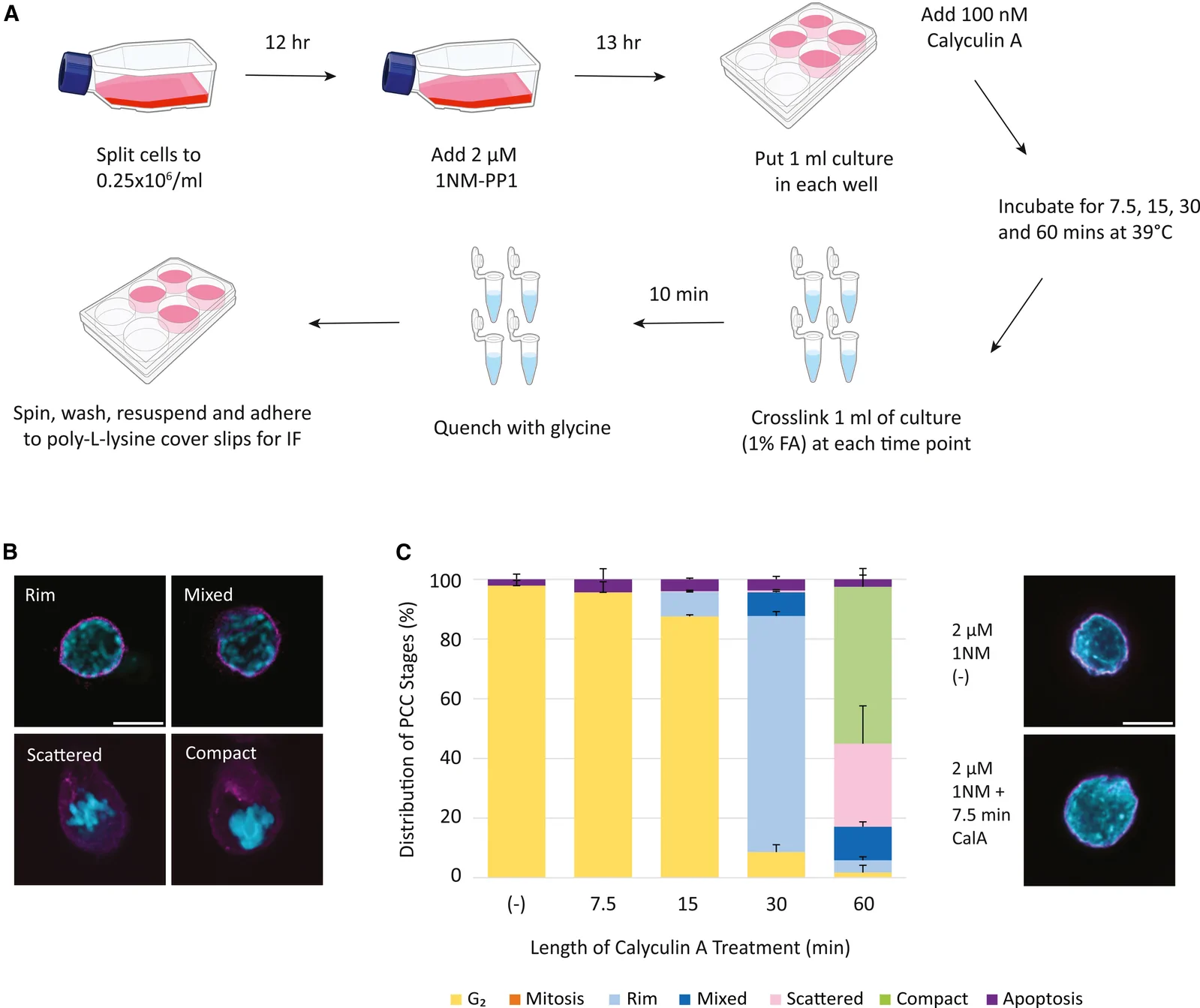
Identification of PCC phenotypes (A) A schematic of PCC induction protocol as described in STAR Methods. (B) Deconvolved immunofluorescence images depicting the four stages of PCC. DNA is shown in cyan and Lamin B1 is in magenta. Scale bars, 5 μm. (C) Distribution of PCC stages across different lengths of calyculin A treatment. Negative (−) control contains G_2_ arrested cells without calyculin A. The bars represent the mean across 2 replicates ±standard deviation. Deconvolved immunofluorescence images showing two exemplary cells from the negative control and the 7.5 min calyculin A treatment. DNA is shown in cyan and lamin B1 is in magenta. Scale bars, 5 μm.

It was unclear, initially whether calyculin A treated CDK1^as^ cells were undergoing a mitotic-like compaction independent of cell cycle progression, or whether the treatment had triggered a premature entry into a canonical mitosis that would culminate in cell division. To test this, we performed a time course with calyculin A and observed that, by 4 h, the chromosomes had become fully compact and clustered, but no division occurred (Figure S1A; Table S1). We conclude that calyculin A treatment of G_2_-blocked CDK1^as^ cells functionally separates chromosome condensation from mitotic progression. This allowed us to investigate the pathway generating condensed chromosomes independent of other aspects of mitosis.

### CDK2 might drive PCC when CDK1 is blocked

The preceding experiments accumulated CDK1^as^ cells in G_2_ using a standard concentration of 1NM-PP1 (2 μM) that allows efficient progression of cells into mitosis upon drug washout. To investigate whether PCC of these cells in the presence of calyculin A required a low residual activity of CDK1^as^ in the presence of 1NM-PP1, we increased the 1NM-PP1 concentration by 5-fold (10 μM). This resulted in a decrease in the efficiency of PCC progression to later stages, with very few cells exhibiting a “scattered” phenotype. However, after 30 min treatment with calyculin A, the vast majority of cells still exhibited a “rim” phenotype with chromatin condensation occurring around the nuclear periphery (Figure 2A).

**Figure 2:**
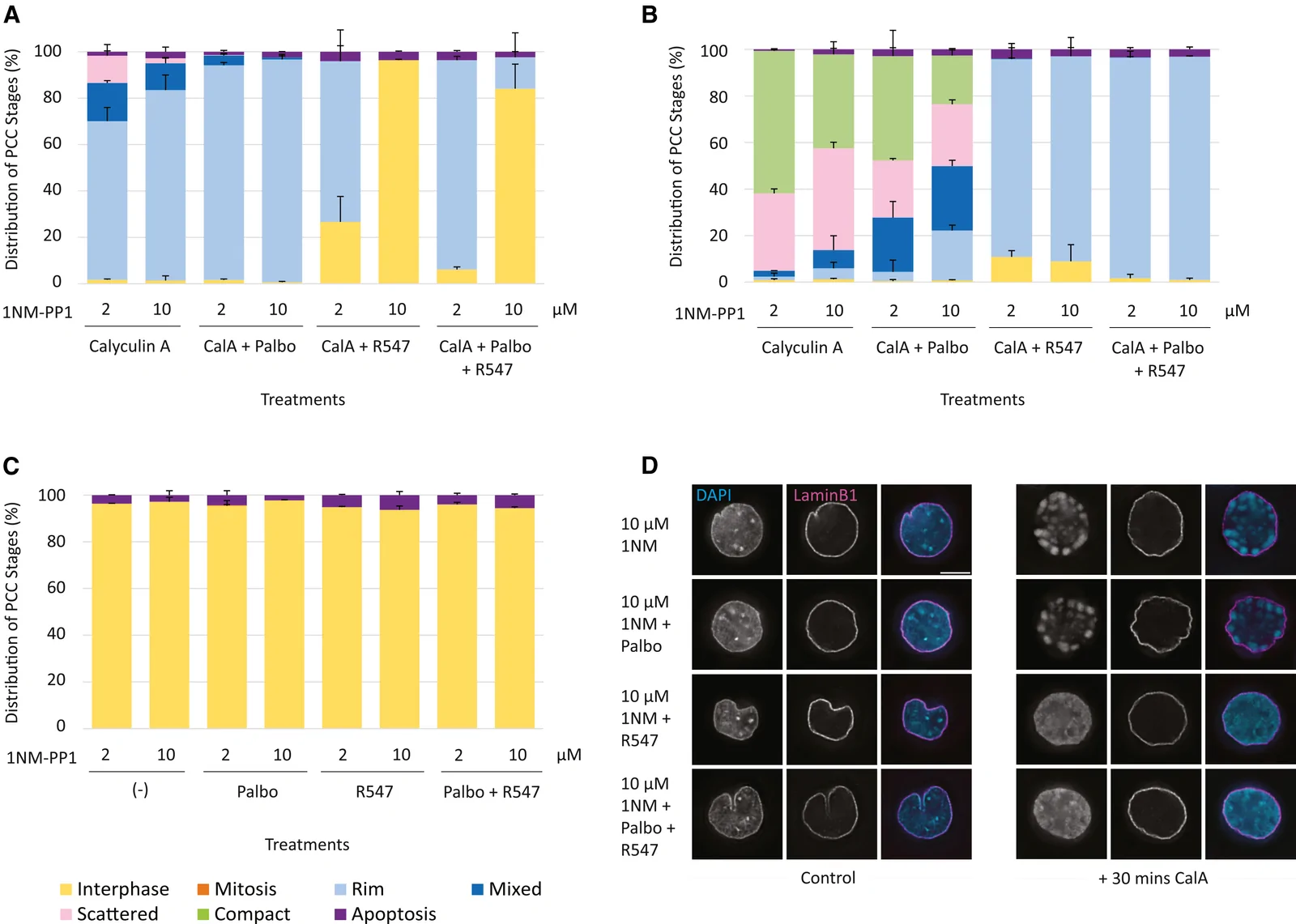
Quantification of PCC phenotypes under different CDK blockers and different lengths of calyculin A treatment. Cells treated with either 2 μM or 10 μM 1NM-PP1 with 4 different additional drug combinations as shown (A) Distribution of PCC stages at 30 min of calyculin A with Palbociclib and/or R547. The bars represent the mean ± standard deviation (*N* = 2). (B) Same as in (A) but at 60 min of calyculin A treatment. (C) Control treatments all of which are arrested in G_2_ for a total of 14 h but lack calyculin A. (D) Deconvolved immunofluorescence images from the experiment with 10 μM 1NM-PP1 showing an example cell for all the controls as well as 30 min calyculin A treated samples with the additional CDK blockers. DNA is shown in cyan and Lamin B1 is in magenta. Scale bars, 5 μm.

Previously, overexpressed CDK2 had been shown to drive mitosis in CDK1-depleted cells.^19^ To ask whether CDK2 or CDK4/6 might drive the “rim” phenotype chromosome condensation in the absence of CDK1 activity, we investigated the impact of R547, a CDK1, 2, and 4 inhibitor, and palbociclib, a CDK4/6 inhibitor, on PCC in the presence of low and high concentrations of 1NM-PP1 plus calyculin A. The experiment consisted of eight combinations of drug treatments, combined with 30 or 60 min of calyculin A (Figures 2A and 2B respectively; Table S1). Note that none of these treatments causes PCC in the absence of calyculin A (Figure 2C). For phenotypes of the various treatments in controls and after 30 min, see Figure 2D.

Cells treated with 2 μM 1NM-PP1 and 30 min of calyculin A gave an average of 68 ± 6% of the cells in “rim” phenotype, in line with the initial set of experiments (Figure 2A). This increased to 82 ± 6.5% when cells were treated with 10 μM 1NM-PP1 and the “scattered” phenotype substantially decreased from 12 ± 5% to only 2.1 ± 0.3%. When palbociclib was added, the rim phenotype made up more than 90% of the samples and the “mixed” phenotype 1%–4% for both concentrations of 1NM-PP1 at 30 min, suggesting a slight delay in chromosome condensation (Figure 2A). In the presence of R547, calyculin A, and 2 μM 1NM-PP1, there were 27 ± 11% of G_2_ cells and 69 ± 13.5% of rim cells indicating a stronger effect than palbociclib. Strikingly, chromosome condensation was fully blocked (96 ± 0.3% G_2_, 4 ± 0.3% apoptosis) at 30 min with 10 μM 1NM-PP1, calyculin A, and R547 (Figures 2A and 2D). The inhibition of PCC was similar when all four drugs were combined. We did not observe a prominent difference in nuclear diameter between the G_2_ cells in negative control (−) and the G_2_ cells in 30 min of calyculin A treatment with R547 block, implying the same degree of overall chromatin compaction between these two conditions (Figure S1B). These results suggest that there is some residual CDK1 activity in the presence of 2 μM 1NM-PP1 and that this is reduced or abolished when the 1NM-PP1 concentration is increased to 10 μM.

A similar trend was seen at 60 min after addition of calyculin A (Figure 2B). In cultures treated with calyculin A in the presence of 2 μM 1NM-PP1, most cells (94.5%) had undergone advanced PCC, with scattered and compact condensed chromosomes and disassembled nuclear lamina. When 1NM-PP1 was increased to 10 μM, the number of cells in advanced PCC (“scattered” and “compact” with disassembled lamin B1) decreased to 84% with a corresponding increase in the number of “rim” and “mixed” cells in early PCC. Addition of palbociclib at both 1NM-PP1 concentrations caused a further shift from late PCC to early PCC phenotypes. Also, a minority of compact cells upon palbociclib addition were observed to have preserved nuclear lamina. Remarkably, the addition of R547 completely abolished the transition into advanced PCC at 60 min and no fully condensed mitotic-like chromosomes were observed. Addition of the three drugs at 60 min yielded a population of nearly 100% of cells at the “rim” stage of early PCC.

Together, these inhibitor treatments suggest that although CDK1 is apparently the usual driver of mitotic-like PCC, CDK2 might substitute to drive PCC when CDK1 is inactive. Under these conditions CDK4/6 also appear to exhibit a limited role in premature chromosomal condensation.

### Calyculin A drives massive phosphorylation of histone H3

In order to identify whether significant changes in histone modifications accompany chromosome condensation following calyculin A treatment, histones were purified by acid extraction from a CDK1^as^ cell culture treated with 10 μM 1NM-PP1, calyculin A, and R547 (Figure 3A).^5^^,^^28^^,^^60^^,^^61^ Importantly, this method preserves phosphorylation marks on histones. When the histones were run on 18% SDS-PAGE, we observed a band shift for histones H3 and H2A on all calyculin A treated samples compared to those from asynchronous cells or cells arrested at G_2_ with 1NM-PP1 ± R547, and without calyculin A (Figure 3B). As in previous experiments (Figure 2; Table S1), cells treated with 10 μM 1NM-PP1, R547, and calyculin A were all in G_2_ phase at 30 min with a negligible (1%) amount of “rim” cells recorded on average across four replicates (Figures 3C; Table S1). After 60 min in calyculin A plus R547, 83% of the cells were in rim phase and 13% were in G_2_ phase.

**Figure 3:**
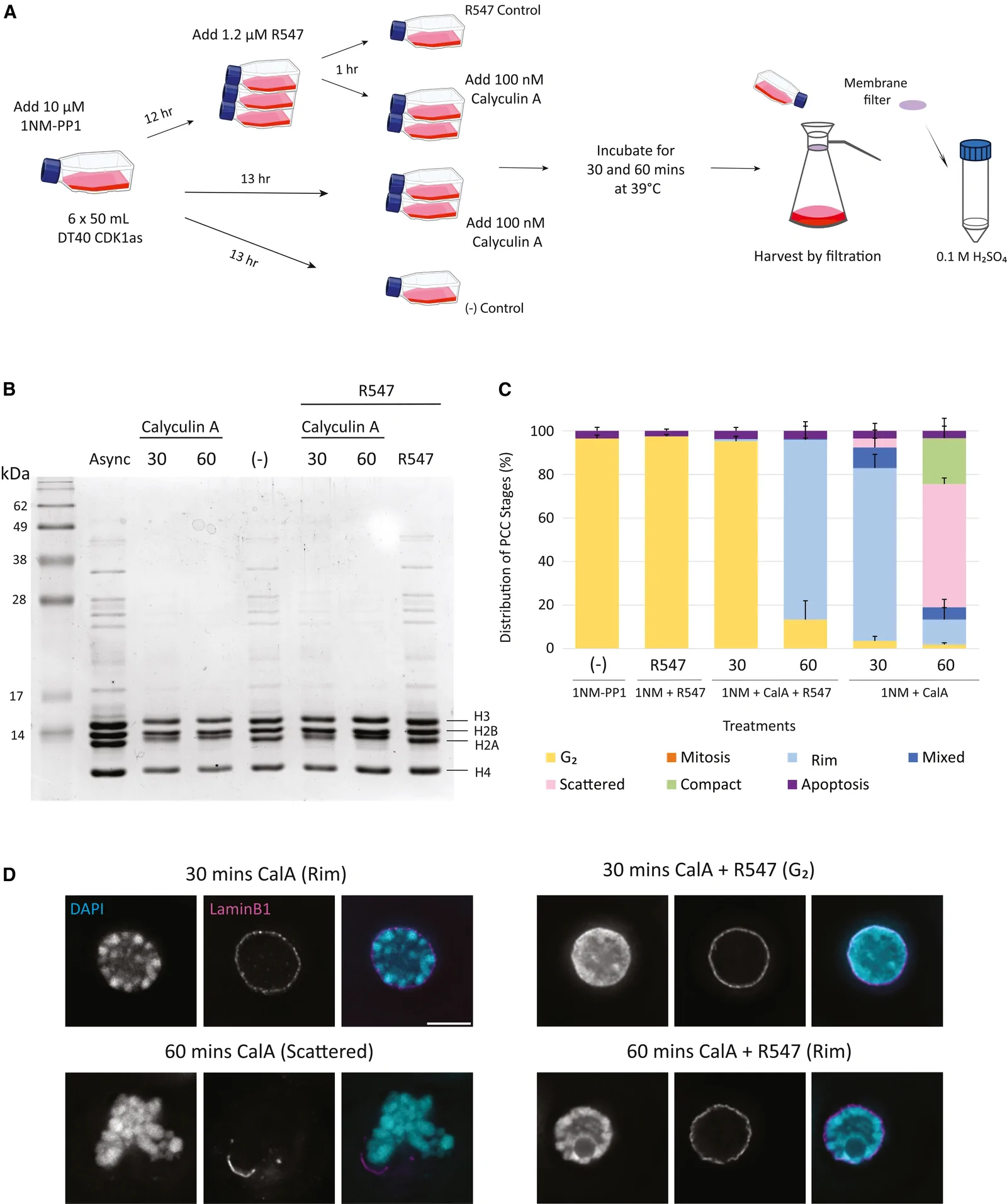
Histone purification from PCC cells (A) A schematic of PCC experiment and histone extraction. (B) Coomassie Blue stained SDS-PAGE gel of purified histone proteins from the experiment. “Async,” histones purified from asynchronous cells. “Calyculin A, 30 or 60 min,” histones purified from cells treated with 10 μM 1NM-PP1 and (optionally) with calyculin A for denoted time. “R547 Calyculin A -, 30 or 60 min,” the same, but pre-treated with R547 before calyculin A addition. (C) Distribution of PCC stages across different drug combinations. Negative (−) control contains G_2_ arrested cells without calyculin A and R547 contains G_2_ arrested cells treated with R547 but not calyculin A. All cells were treated with 10 μM 1NM-PP1. The bars represent the mean across 4 replicates ±standard deviation. (D) Deconvolved immunofluorescence images of all calyculin A treatment conditions with the addition of R547. DNA is in cyan and Lamin B1 is in magenta. Scale bars, 5 μm.

As shown previously, PCC progressed further in the absence of R547 (Figures 3C and 3D). At 30 min, most cells (79%) showed a “rim” phenotype with lesser amounts of “mixed” (9%) and “scattered” (4%) phenotypes. By 60 min, most (78%) of the cells had progressed further to form fully condensed chromosomes (“scattered” and “compact” phenotypes) with the remainder corresponding to “mixed” and “rim” phenotypes.

The histone modification status was determined for these samples by LC-MS^28^^,^^35^^,^^62^ (Table S2). We focused on the 30-min sample in the presence of calyculin A plus R547. This sample contained only G_2_ cells lacking condensed chromosomes, despite SDS-PAGE indicating the presence of modifications of core histones (indicated by the band shifts of H3 and H2A; Figure 3B). The following analysis focuses on histone H3. Mass spectrometry analysis of histone H2A is very challenging, and the cause of the band shift in H2A observed by SDS-PAGE remains to be determined.

### Calyculin A treated cells exhibit much more efficient histone H3 phosphorylation than mitotic chromosomes despite a lack of chromatin condensation

Our analysis initially focused on histone phosphorylation as calyculin A is an inhibitor of protein phosphatases. Previously, measurements in late prometaphase (30 min after release from 1NM-PP1 block in free-running mitosis) detected approximately 90% efficiency of phosphorylation on Ser10, and approximately 25%–35% efficiency of phosphorylation on T3 of histone H3 and Ser28 of histone 3.1.^28^

Cells blocked with 10 μM 1NM-PP1 and treated with calyculin A for 30 min exhibited 96 ± 1% of peptides phosphorylated on serine 10 of histone H3 (Figure 4A). By 60 min, 97 ± 1% of Ser10-containing peptides from H3 were phosphorylated. This contrasts with 89 ± 1% of peptides containing H3S10ph in prometaphase chromosomes at 30 min into free-running mitosis after release from a 1NM-PP1 block.^28^ Importantly, cells treated with R547 prior to calyculin A treatment also showed extraordinary levels of phosphorylation of H3S10 (95 ± 2% - Figure 4A). This is remarkable because at 30 min of calyculin A treatment with R547 no condensed chromatin was observed (Figures 3C and 3D).

**Figure 4:**
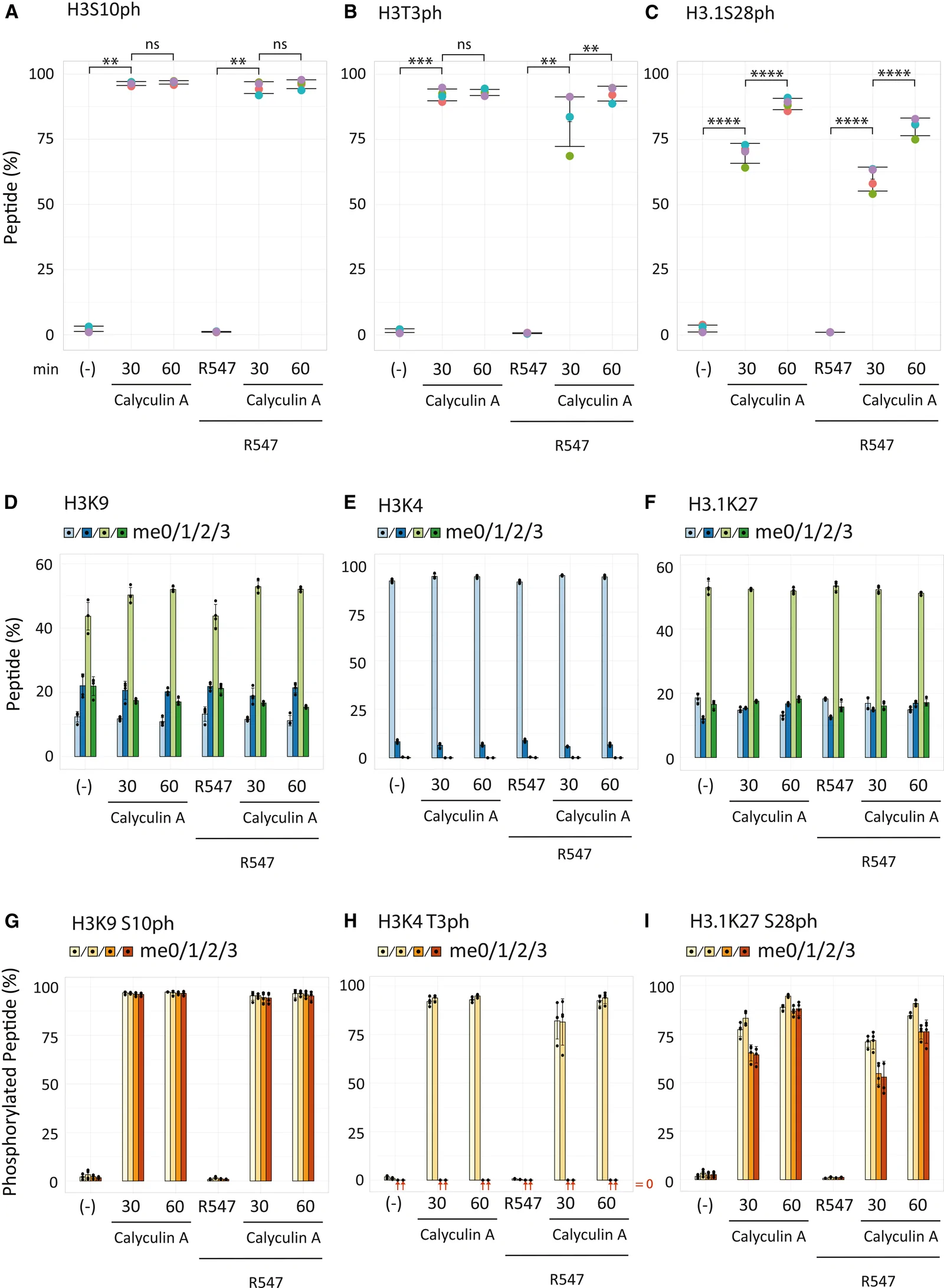
Three different phosphorylation events throughout calyculin A treatment (A–C) H3S10, H3T3, and H3.1S28 phosphorylation levels out of the total peptide in percentage. Individual replicates are shown with distinct color dots (*N* = 4). Bars represent mean ± standard deviation. For statistical comparison, Kruskal-Wallis test, followed by Conover-Iman test (in case of *p* < 0.05 in Kruskal-Wallis test) was performed. The *p* values were adjusted with Benjamini-Hochberg procedure. (D) A grouped barplot of the total mean peptide percentage of H3K9me0, me1, me2, and me3 and modifications under calyculin A and/or R547 treatment. Bars represent mean ± standard deviation (*N* = 4). (G) A grouped barplot of the phosphorylated H3K9me0, me1, me2 and me3 percentage (percentage of total peptides which are phosphorylated normalized to the total peptide percentage of the respective modifications). Bars represent mean ± standard deviation (*N* = 4). (E and H) The same as in (D and G), but for H3K4me0, me1, me2, and me3. (F and I) The same as in (D and G) or (E and H), but for H3.1K27me0, me1, me2, and me3.

Surprisingly, phosphorylation levels on threonine 3 of histone H3 also reached 92 ± 2% at 30 min and 93 ± 1% by 60 min of calyculin A (Figure 4B). This was vastly higher than levels of H3T3 phosphorylation in free-running mitosis, which peaked at 29% in early prometaphase chromosomes at 15 min after release from a 1NM-PP1 block.^28^ Pretreatment of cells with R547 reduced H3T3 phosphorylation levels only slightly (82 ± 10% at 30 min and 93 ± 3% at 60 min) despite completely abolishing visible chromatin condensation.

Phosphorylation of serine 28 on histone H3.1 was relatively delayed compared to phosphorylation of T3 and S10 (Figure 4C). This agrees with the trend observed in free-running mitosis,^28^ however once again phosphorylation levels were much higher in cells treated with calyculin A. At 30 min of calyculin A treatment H3S28 phosphorylation reached 70 ± 4% and by 60 min levels had risen to 89 ± 2%. These values were decreased only slightly in cells treated with R547 (60 ± 5% at 30 min and 80 ± 3% by 60 min in calyculin A). Once again, these values are much higher than the ∼30% seen in free-running mitosis.^28^

Together, these results reveal that in calyculin A-treated cells where CDK1 and CDK2 have been inhibited, histone H3 phosphorylation is uncoupled from chromosome condensation.

### Phosphorylation levels show selective associations with methylation marks

Many studies of gene expression have focused on the methylation of histone H3 lysine 4 (H3K4). For example, H3K4me2 and H3K4me3 show a significant association with promoters. What is often not appreciated is that these two marks together comprise less than 1% of the peptides containing K4. In contrast H3K4me0 and H3K4me1 make up >90% and 6%–9% of the corresponding peptide, respectively (Figure 4E;^28^). This variability between the abundance of methylation states or their absence is seen less in the case of H3K9me and H3K27me (Figures 4D and 4F; Kochanova et al.^28^). Our previous study found that H3K4me0 and H3K4me1 show similar levels of phosphorylation on T3—up to 30%. In marked contrast, peptides containing H3K4me2 and H3K4me3 were completely devoid of T3 phosphorylation. We therefore suggested that T3 phosphorylation might be absent from promoters in active euchromatin.

Our present studies suggest that this explanation is not sufficient. Firstly, we quantitated the levels of the various methylation states of H3K4 in free-running mitosis and in cells treated with calyculin A ± R547. As in our prior study, we found no significant changes upon entry into free-running mitosis,^28^ but it should be noted that levels of H3K4me2 and H3K4me3 are so low (<1%) that measurements of differences may not be significant. The slight differences between control and calyculin A treated samples (Figures S2A and S2B) could be consistent with transcriptional shutdown in the presence of calyculin A based on the known association of H3K4me2/3 with promoters.^63^

As in our previous study, we observed an absolute lack of detectable T3 phosphorylation on peptides containing H3K4me2 and H3K4me3 (Figure 4H). In stark contrast, 93%–95% of H3 peptides containing H3K4me0 and H3K4me1 were phosphorylated in cells treated with calyculin A for 60 min (Figures 4B and 4H). This contrasts with a maximum of 30% in cells undergoing free-running mitosis.^28^ The lack of T3 phosphorylation of peptides containing H3K4me2 and H3K4me3 in calyculin A-treated cells is unlikely be because those peptides are found only in euchromatin, as under these conditions >90% of chromatin is phosphorylated on T3. However, we cannot exclude that the histone H3 molecules containing H3K4me2 and H3K4me3 are exclusively found in the small percentage of H3 that is not phosphorylated on T3.

The two other predominant phosphorylated residues on histone H3 (S10 and S28) also sit next to methylated residues, K9 and K27, which bind factors involved in constitutive and facultative heterochromatin, respectively (HP1 and polycomb-related proteins).^64^^,^^65^^,^^66^ These peptides show no methylation-dependent difference in phosphorylation efficiency with H3K9me0-me3 peptides all showing 86%–91% phosphorylation of S10 at 30 min of free-running mitosis^28^ and 94%–97% phosphorylation in calyculin A-treated cells either with or without R547 pretreatment (Figure 4G). Similarly, H3K27me0-me3 peptides show lower (and temporally delayed) 27%–45% phosphorylation of S28 at 30 min of free-running mitosis and exhibit a temporally delayed 53%–95% phosphorylation in calyculin A-treated cells either with or without R547 pretreatment (Figure 4I). Notably, high levels of S10 and S28 phosphorylation were observed in cells pretreated with R547 at 30 min after addition of calyculin A, at which time no evidence of chromosome condensation is observed (Figure 3D).

### Histones are gradually deacetylated with increasing exposure to calyculin A

Several studies have suggested that histone deacetylation may help to drive mitotic prometaphase chromosome compaction.^2^^,^^4^^,^^5^^,^^33^ However, we recently found that histone acetylation levels diminish only slightly during mitotic entry in a free-running mitosis, whereas they fall precipitously if the cells are blocked in prometaphase with drugs that block mitotic progression.^28^

In order to investigate histone acetylation in the presence of calyculin A and R547, we measured the acetylation levels of selected peptides in corresponding histones under the different conditions. Monoacetylation on H2AK5/K9 and H3K9/K14, as well as diacetylation on H4K5/K8/K12/K16 all were significantly decreased at 30 min of calyculin A and further decreased by 60 min (Figures 5A–5C). This trend was very similar when R547 was added prior to calyculin A treatment, where the same general pattern was observed across a number of acetylated peptides: a slight, but significant, decrease in acetylated peptides after 30 min of calyculin A treatment and a more prominent decrease after 60 min of calyculin A (Figure 5D). We conclude although the significant levels of histone deacetylation observed at 60 min of calyculin A treatment in the presence of R547 could be associated with low levels of “rim” chromatin compaction, they are not sufficient to induce formation of fully condensed chromosomes in cells.

**Figure 5:**
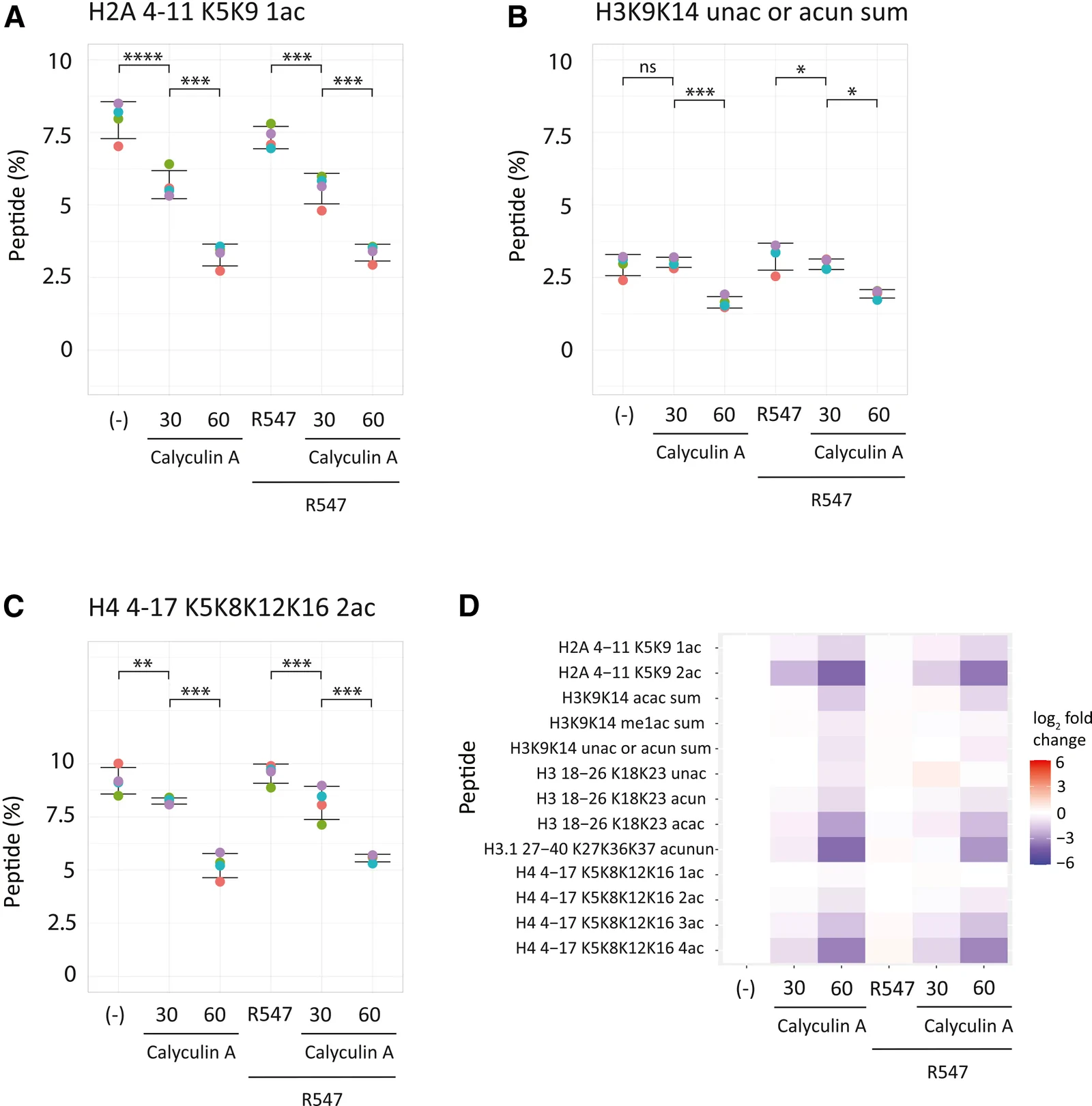
Acetylation levels under calyculin A and/or R547 treatment (A–C) H2A K5K9 1ac, H3 K9K14 unac or acun and H4 K5K8K12K16 2ac levels out of the total peptide in percentage. Individual replicates are shown with distinct color dots (*N* = 4). Bars represent mean ± standard deviation. For statistical comparison, Kruskal-Wallis test, followed by Conover-Iman test (in case of *p* < 0.05 in Kruskal-Wallis test) was performed. The *p* values were adjusted with Benjamini-Hochberg procedure. (D) A heatmap representing mean log_2_fold change of acetylation levels of histones H2A, H3, and H4 as relative to G_2_. Peptides denoted as “sum” were not measured as a whole, but represent the sum of measured % of all their ph and non-ph versions.

### Condensin localizes to prematurely condensed chromosomes

Condensins are molecular motors that are required for the formation of rod-shaped mitotic chromosomes.^17^^,^^67^^,^^68^ What is less clear is the link between condensin activity and the program of chromatin phosphorylation of mitotic chromosomes. To investigate this link, we imaged fluorescently labeled SMC2 (a subunit of both condensin I and II) in cells following the various treatments described above. Cells with a G_2_-like DNA distribution showed diffuse condensin localization with condensin predominantly localizing to the cytoplasm. In contrast, condensin was significantly enriched on prematurely condensed chromosomes that had progressed to achieving a rod-shaped morphology. Importantly condensin was never detected on chromatin and indeed remained largely in the cytoplasm in all experiments where R547 was added (Figure 6). Together, these experiments indicate that CDK activity is required for condensin to efficiently enter the cell nucleus and associate with chromatin.

**Figure 6:**
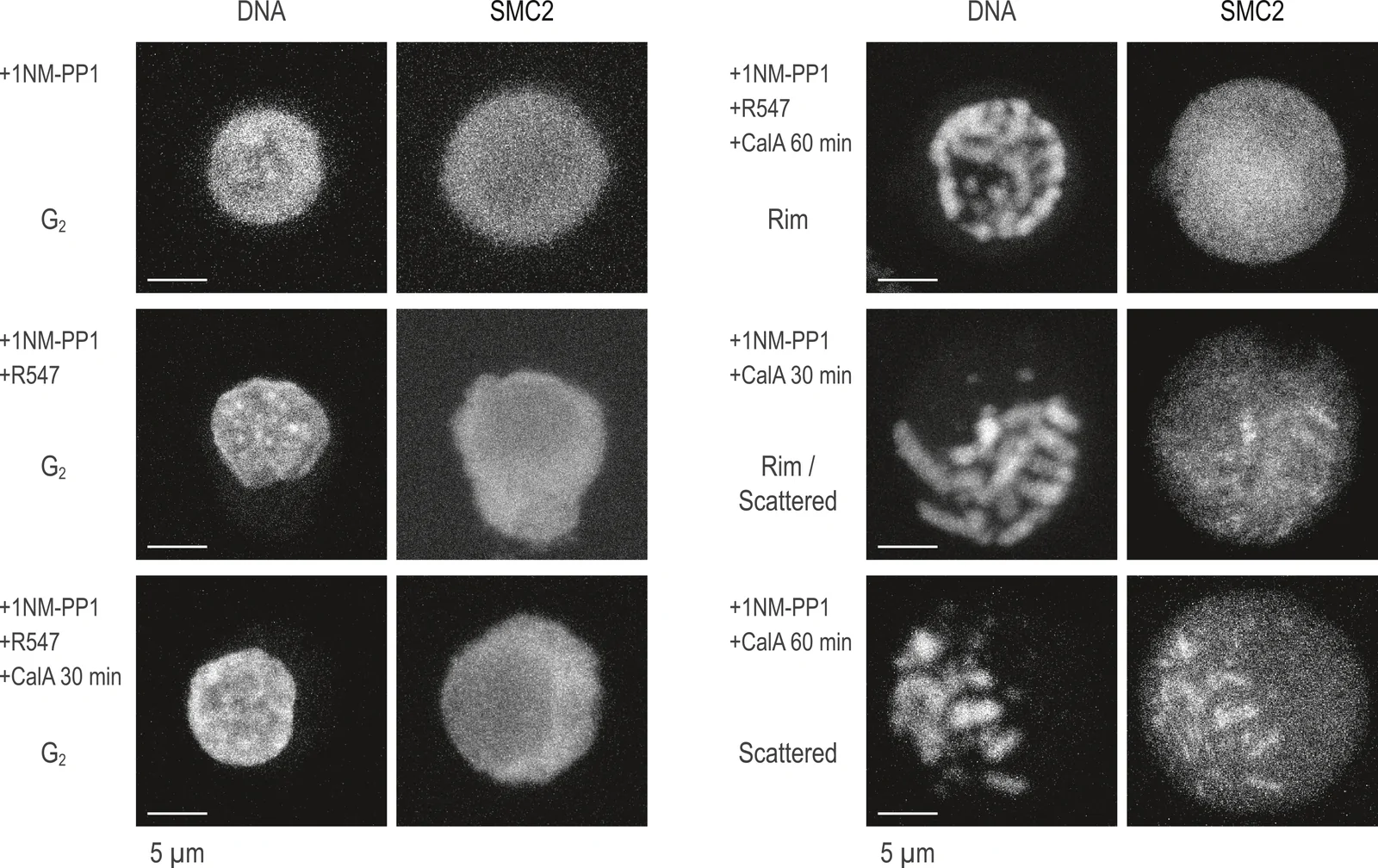
Condensin localizes to prematurely condensed chromosomes Immunofluorescence images (maximum intensity projections) of DT40 chicken cells with DNA stained with DAPI and SMC2-Halo labeled with JFX549 for indicated drug treatment conditions. Scale bars, 5 μm.

We conclude that in cells treated with calyculin A, the phosphorylation of histone H3 achieves the same remarkably high levels regardless of whether or not condensins are concentrated on the chromosomes. Thus condensin binding is not required for mitotic-like chromatin phosphorylation and conversely, that histone H3 phosphorylation is not sufficient to drive condensin targeting to the chromosomes.

## Discussion

We describe a system to efficiently induce mitotic-like chromosome condensation in cells in which endogenous CDK1 alleles have been replaced by analog-sensitive CDK1^as^ alleles that can be specifically inhibited with 1NM-PP1. Treatment of these cells with 1NM-PP1 causes an efficient arrest in late G_2_ that can be reversed upon drug washout. Here, we show that addition of calyculin A, an inhibitor of protein phosphatases 1 and 2A, causes these cells to undergo a pathway resembling mitotic prophase and to eventually acquire a prometaphase-like phenotype with PCC and a disassembled nuclear lamina, all in the presence of 1NM-PP1. This is apparently not a forced entry into canonical mitosis, as cells fail to divide within 4 h. The failure of mitosis to progress might reflect the efficient inhibition of CDK1^as^ with 1NM-PP1, since CDK1 is reportedly indispensable for anaphase and cytokinesis.^19^

Although 1NM-PP1 causes a highly efficient cell-cycle arrest in late G_2_, it appears that some CDK1 activity remains, as increasing the 1NM-PP1 concentration 5-fold to 10 μM caused a decrease in chromosome condensation at early time-points (but did not block the process). Importantly, in cells treated with 10 μM 1NM-PP1 addition of R547, an inhibitor of CDK1, CDK2, and CDK4,^69^ abolished PCC after calyculin A treatment, with nuclei retaining an interphase appearance. Importantly, some peripheral chromatin compaction was seen after 60 min exposure to calyculin A. Treatment of these cells with the CDK4/6 inhibitor palbociclib had only minor effects on the PCC phenotypes observed here. As R547 is a relatively selective inhibitor of CDK2 and CDK4 (it was also reported to inhibit CDK7 and some PCTK kinases in one study^70^), our data thus suggest that calyculin A-induced PCC in CDK1^as^ cells is likely driven by residual activity of CDK1 with additional contributions from CDK2 (and possibly CDK4 to a lesser extent).

LC-MS analysis revealed that in cells undergoing PCC (1NM-PP1 + calyculin A), the phosphorylation efficiency of histone H3 peptides containing threonine 3, serine 10, and serine 28 approaches 100%. This is in striking contrast to free-running mitosis, where the maximum values are ∼30%, ∼90%, and ∼30%, respectively.^28^ This remarkable increase in T3 and S28 phosphorylation could either be because those residues are normally fully phosphorylated, but then efficiently de-phosphorylated by PP1 and PP2A, or because inhibition of those two phosphatases increases the efficiency of haspin phosphorylation of H3T3^38^^,^^71^ and aurora B phosphorylation of H3S10 and H3S28.^41^^,^^71^^,^^72^

Strikingly, levels of histone Hyper-phosphorylation remained nearly as high in G_2_-arrested cells that were pretreated with R547 prior to 30 min in calyculin A. Under these conditions, chromosome condensation is essentially prevented and cells remain in interphase. This clearly shows that histone phosphorylation per se is not sufficient for the formation of rod-shaped condensed mitotic-like chromosomes. However, we note that this does not exclude the possibility that histone phosphorylation does contribute to the formation of condensed mitotic chromosomes.

Likewise, histone deacetylation, which has been proposed to drive mitotic chromosome compaction,^2^^,^^4^^,^^5^^,^^33^ occurs in calyculin A-treated cells in both the absence and presence of R547. In the latter cells (calyculin A + R547) chromatin condensation is abolished at 30 min and severely inhibited at 60 min (where only an early “rim” phenotype is observed). Thus, it appears that histone deacetylation is not sufficient to drive mitotic-like chromosome condensation.

A remarkable aspect of histone H3 primary sequence is that lysine residues whose methylation status influences the binding of chromatin reader proteins all reside immediately adjacent to serine or threonine residues that are phosphorylated in mitosis. This led to the influential methyl-phos switch hypothesis when it was revealed that the phosphorylation of Serine 10 can displace heterochromatin protein HP1 bound to H3K9me3.^73^^,^^74^ Similar observations were reported for H3K27^75^^,^^76^ and H3K4,^77^^,^^78^^,^^79^^,^^80^ although the observation that peptides containing H3K4me2/3 are seldom phosphorylated called into question this mechanism^29^^,^^81^ for H3K4me2/3, at least. Rigorous quantitative LC-MS analysis revealed that T3 phosphorylation is completely undetectable on peptides containing H3K4me2 and K4me3.^28^^,^^29^ In contrast, normal levels of T3 phosphorylation were observed on peptides containing H3K4me0 and H3K4me1.

In cells treated with calyculin A, we find that levels of H3T3 phosphorylation rise to nearly 100%. Thus, in these cells, T3ph is no longer a specific marker for heterochromatin. Despite this finding, we still failed to detect any phosphorylation of peptides containing H3K4me2 and K4me3. Because multiply modified and hydrophilic peptides can suffer from low ionization efficiency, poor retention, or technical detection limits in mass spectrometry, non-detection alone does not definitively prove absence. However, following calyculin A treatment, we observed no measurable depletion in the levels of unphosphorylated H3K4me2 and H3K4me3 peptides. Had these species been phosphorylated at rates comparable to unmodified or monomethylated H3, a corresponding decline in the unphosphorylated H3K4me2/3 pools would be expected. Together, these observations favor a working hypothesis in which higher-order methylation on lysine 4 inhibits threonine 3 phosphorylation *in vivo*, in accordance with previous results of Harris et al.^81^ This is consistent with structural data showing that di- and tri-methylation of K4 physically occludes access to the haspin catalytic pocket.^82^^,^^83^^,^^84^^,^^85^ An alternative possibility is that binding of a specific reader to H3K4me2 and K4me3 interferes with haspin binding.

The results reported here together with our previous finding that histone deacetylation was minimal during free-running mitotic entry up to the stage of late prometaphase -^28^ reveal that neither histone phosphorylation nor deacetylation is sufficient for mitotic chromatin compaction. This is consistent with results of previous studies of mitotic chromosome mechanics following HDAC inhibitor treatment.^86^ As far as we are aware, the present results leave two proposed mechanisms contributing to this compaction: an increase in Mg^2+^ concentration produced by ATP hydrolysis^87^ and a rise in molecular crowding due to increased density of proteins, RNAs and other molecules in the mitotic cytoplasm.^88^ This therefore remains a fruitful area for future research.

### Limitations of the study

Our system allows us to to uncouple chromosome condensation from mitotic progression. However, the inhibition of phosphatases by calyculin A leads to a non-physiological hyper-phosphorylation of core histone which might have a potential effect on other histone modifications measured. Furthermore, changes in phosphorylation of other chromosomal proteins cannot be excluded. In this study, we use chicken DT40 B lymphocytes, and we acknowledge that histone modification patterns might differ in other species or cell types. Moreover, due to LC-MS limitations, we were unable to unambiguously detect phosphorylation on H2AT120 and H3.3S28/S31, as well as acetylation of histone H2B. In addition to this, LC-MS experiments were performed without spike-in peptides, which makes our quantification approximate.

## Resource availability

### Lead contact

Further information and requests for resources and reagents should be directed to and will be fulfilled by the lead contact, Axel Imhof (imhof@lmu.de).

### Materials availability

The SMC2-Halo CDK1^as^ DT40 cell line is available upon request from the Earnshaw lab.

### Data and code availability

• Histone mass spectrometry data are deposited to the ProteomeXchange Consortium via the PRIDE partner repository: PXD078615.
• Immunofluorescence and raw gel data are deposited to Mendeley Data: https://data.mendeley.com/datasets/txfcb7wpps/1.
• The R code used to generate plots is available upon request.
• Any additional information required to reanalyze the data reported in this study is available from the lead contact upon request.

## Acknowledgments

We thank Samuel Corless for manuscript draft editing and Maria Alba Abad for advice on histone preparation. We thank Shaun Webb from the Bioinformatics core, as well as Antoni Sieminski and the Center for Statistics Drop-in Clinic at the University of Edinburgh for help with statistics. We thank Edinburgh Genome Foundry for cloning recombination template which was used for SMC2-Halo cell line generation. We also acknowledge NIH BioArt for using their images in figures. This work was funded by 10.13039/100004440Wellcome grants 107022 and 221044 to WCE, as well as 203149 to the Wellcome Center for Cell Biology. This work was also supported by funding for the Wellcome Discovery Research Platform for Hidden Cell Biology [226791] and we gratefully acknowledge support from the Bioinformatics core. AI lab was funded via grants from the 10.13039/501100001659Deutsche Forschungsgemeinschaft (DFG, 419067076 and 213249687).

## Author contributions

I.U., M.B., N.Y.K., I.F., C.R., and K.S. performed the experiments; I.U., M.B., N.Y.K., C.R., and B.P. analyzed the data; L.X. and J.R.P. synthesized 1NM-PP1; W.C.E., A.I., and N.Y.K. lead the project; W.C.E. and A.I. acquired funding; I.U., N.Y.K., W.C.E., and A.I. wrote and edited the study with input from all co-authors.

## Declaration of interests

The authors declare no conflict of interest.

## STAR★Methods

### Key resources table

**Table:** 

REAGENT or RESOURCE	SOURCE	IDENTIFIER
**Antibodies**		

Rabbit anti-LaminB1, batch 1022148–1	Abcam	Cat#ab16048
Goat anti-Rabbit IgG Alexa Fluor™ Plus 488	Thermo Fisher Scientific	Cat#A32731
Goat anti-Rabbit Alexa Fluor™ 555	Thermo Fisher Scientific	Cat#A32732

**Chemicals, peptides, and recombinant proteins**		

Chicken serum	Thermo Fisher Scientific	16110082
1NM-PP1	Laboratory of James Paulson	N/A
Calyculin A	Tocris Bioscience	Cat#1336
Calyculin A	Thermo Fisher Scientific	Cat#PHZ1044
R547	MedChemExpress	Cat#HY-10014
Palbociclib	Selleckchem	Cat#PD-0332991
Halo tag dye (JFX549)	Laboratory of Luke Lavis	N/A
Hygromycin	Roche	Cat#10843555001
Sulfuric acid (H_2_SO_4_)	Sigma-Aldrich	Cat#258105
Phenylmethylsulfonyl fluoride (PMSF)	Sigma-Aldrich	Cat#P7626
DTT	Melford	Cat#D11000–25.0
Perchloric acid 70%	Fisher Scientific	Cat#10117900
Hydrochloric acid (HCl)	Fisher Scientific	Cat#10053023
Acetone	Fisher Scientific	Cat#11433733
Coomassie Imperial™ Protein Stain	Thermo Fisher Scientific	Cat#24615
Bovine serum albumin (BSA)	Sigma-Aldrich	Cat#A7284
Poly-L-Lysine solution (0.1% in water)	Sigma-Aldrich	Cat#P8920
ProLong™ Glass Antifade Mountant	Thermo Fisher Scientific	Cat#P36980
Vectashield Plus	Vector Laboratories	Cat#H-1900–2
Vectashield Antifade with DAPI	Vector Laboratories	Cat#H-1200–10
Coomassie Instant Blue	Abcam/Expedeon	Cat#ab119211
NH_4_HCO_3_	Carl ROTH	Cat#T871.1
Acetonitrile	Carl ROTH	Cat#AE70.1
Propionic anhydride	Merck	Cat#8.00608.0100
Trypsin	Promega	Cat#V5111
TFA	Thermo Fisher Scientific	Cat#85183
EDTA	Thermo Fisher Scientific	Cat#17892
Thermo Scientific™ Pierce™ 16% Formaldehyde (w/v), Methanol-free	Thermo Fisher Scientific	Cat#11586711
Methanol	Fisher Scientific	Cat#11976961
Glacial Acetic Acid	Fisher Scientific	Cat#A/0400/PB17
Glycine	Sigma-Aldrich	Cat#G8790
20% SDS	Thermo Fisher Scientific	Cat#AM9820
Triton X-100	Sigma-Aldrich	Cat#X100
UltraPure™ Tris Buffer	Thermo Fisher Scientific	Cat#15504020
NaCl	Fisher Scientific	Cat#10428420
KCl	Fisher Scientific	Cat#11459133
DTT	Thermo Fisher Scientific	Cat#R0861
DPBS (1X)	Gibco	Cat#14190–094

**Deposited data**		

Histone mass spectrometry data	This study	PRIDE20 partner repository: PXD078615

**Experimental models: Cell lines**		

CDK1^as^ DT40 cells	Laboratory of Bill Earnshaw, Gibcus et al.^18^	N/A
SMC2-Halo CDK1^as^ DT40 cells	Laboratory of Bill Earnshaw, this study	N/A

**Oligonucleotides**		

SMC2_Halo_GuideRNA_sense: CACCGAGTGAAGCCAGCAACAACA	This study	N/A
SMC2_Halo_GuideRNA_antisense: AAACTGTTGTTGCTGGCTTCACTC	This study	N/A

**Recombinant DNA**		

pX330 with guide RNAs for SMC2	This study; originally Cong et al.^89^	Addgene plasmid # 42230
The plasmid for SMC2-Halo CRISPR/Cas9 with Hygro resistance and homology arms	Each piece of DNA was generated by geneart and assembled^90^	Assembled by genome foundry (University of Edinburgh)

**Software and algorithms**		

DeltaVision softWoRx 7.2.2 Software Release RC1 DeltaVision Instrument Controller 5.40 (build 1076)	Cytiva	N/A
ImageJ	NIH	https://imagej.net/ij/
Image Studio™ Software	LICORbio^TM^	https://www.licorbio.com/image-studio
Skyline	Laboratory of Michael MacCoss, Pino et al.^91^	https://skyline.ms/
Proteome Discoverer 1.4	Thermo Fisher Scientific	https://thermo.flexnetoperations.com/
R environment	R Core Team^92^	https://www.r-project.org/

**Other**		

Nalgene™ Reusable Filter Unit	Thermo Fisher Scientific	Cat#300-4050 PK
0.2 μm nitrocellulose filter disks	Whatman	Cat#7182–004
SP Sepharose^TM^ Fast Flow beads	Cytiva	Cat#17072910
Precast 4–20% polyacrylamide gels (SERVAGel™ TG PRiME™ 4–20%)	SERVA	Cat#43289.01
C8 Empore disk	Sigma/Supelco	Cat#66882-U
25 cm analytical column (75 μm ID, 1.6 μm C18)	Aurora-IonOpticks	Cat#ODY-25075C18A

### Experimental model and study participant details

#### Cell culture

The analogue sensitive CDK1 (CDK1^as^) DT40 chicken lymphocyte cell line^18^^,^^28^^,^^58^^,^^59^ was used in all experiments in this study. Additionally, for the investigation of condensin localisation to PCC, an SMC2-Halo tag cell line was generated and used.^59^ The establishment of DT40 CDK1^as^ cells were described previously.^18^ The DT40 CDK1^as^ cell line was grown in RPMI 1640 medium with 10% fetal bovine serum and 1% chicken serum at 39°C in 5% CO_2_ incubator and diluted daily to 1.5 x 10^5^/mL. The cell line used in this study was grown in the absence of antibiotics and tested negative for mycoplasma contamination. Cells were incubated for 13 h with 2 or 10 μM 1NM-PP1 for G_2_ arrest. After 13 h the cells were treated with 100 nM calyculin A to induce PCC. Cells were additionally treated with 1.2 μM R547 or 24 μM Palbociclib at 12 h incubation of 1NM-PP1 to further block CDKs 1, 2 and 4 and/or CDK4/6.

#### SMC2-halo cell line establishment

The cell line was generated in CDK1^as^, TIR1 overexpressing background^59^ using CRISPR-Cas9. A Halo tag knock-in construct was synthesized by GeneArt and assembled at the C-terminus of the SMC2 gene by the Edinburgh Genome Foundry.^90^ Oligo pairs used to generate a derivative of the pX330 plasmid^89^ (Addgene #42230) were CACCGAGTGAAGCCAGCAACAACA and AAACTGTTGTTGCTGGCTTCACTC. After selection with 0.75 μg/mL hygromycin, individual clones were examined by Western blotting analysis with an anti-SMC2 antibody^93^ and an anti-Halo tag monoclonal antibody (Promega G9211).

### Method details

#### Media, drug treatments and antibodies

Media and serums were obtained from Invitrogen. 1NM-PP1 was synthesised by James R Paulson and Linfeng Xie and dissolved in DMSO at 10 mM. Calyculin A (Invitrogen and Tocris Bioscience) was dissolved in DMSO at 100 μM. Aliquots were stored in −80°C long-term and at −20°C after the first thaw. R547 was obtained from Merck and dissolved at 6 mM in DMSO. Also stored at −80°C.

The antibodies used for indirect immunofluorescence were Rabbit polyclonal anti-LaminB1 (1:1000, ab16048, Abcam) and Alexa Fluor 488 goat anti-rabbit IgG (1:1000, A32731, Invitrogen) or Alexa Fluor 555 goat anti-rabbit IgG (1:1000, A32732, Invitrogen).

#### Indirect immunofluorescence

The procedure was done as described in.^28^ The 1 mL of cells per sample were crosslinked in 1% formaldehyde for 10 min and quenched with 250 μL of 2.5 M glycine. Cells were adhered onto poly-L-lysine coated coverslips, fixed with prewarmed (37°C) 4% formaldehyde for 10 min and permeabilised with 0.15% Triton X-100 in PBS for 5 min at RT with rinses in PBS in between and after Triton treatment. Cells were then blocked with 5% BSA in PBS for 30 min, incubated for 1 h with indicated primary antibody in blocking buffer followed by another 30 min with the secondary antibody in blocking buffer in the dark. Coverslips were washed 2 times for 5 min in PBS. Finally stained with Hoechst 33342 and mounted on microscope slides with VectaShield Plus (Vector Laboratories) or Prolong Glass (Invitrogen). For visualisation of Halo-tagged SMC2, Halo-JFX549 was added to directly to culture media at 1/50,000 dilution (from 1 mM stock concentration), 1 h before fixation. Cells were collected and resuspended in 500 μL pre-warmed (37^o^C) 75 mM KCl and incubated for 5 min before being fixed in 1 mL ice-cold methanol:acetic acid. Cells were then fixed overnight at −20^o^C, before being dropped onto coverslips. After drying, coverslips were mounted using Vectashield + DAPI (Vector Laboratories).

DeltaVision Elite High-Resolution microscope with Olympus UPlanXApo 100X, NA 1.45 or Olympus UPlanXApo 60X, NA 1.42, oil objectives were used to image the cells in all experiments, with the exception of the condensin localisation experiment. Images were deconvolved using Delta Vision SoftWoRx 6.0 software (Applied Precision/GE Healthcare). For condensin localisation, confocal images were acquired using Zeiss LSM 980 confocal microscope with 100× alpha Plan-Apochromat objective/1.4 Oil DIC. The planes were acquired with a 0.17-μm interval. All images were processed using ImageJ software.

#### Histone purification and SDS-PAGE

The procedure was done as described in.^28^ Cell cultures containing 25 to 50 million cells were filtered through Whatman nitrocellulose membrane filter (0.2 μm, 47 mm, Cytiva). The filters were crumpled into a 50 mL tube filled with 5 mL 0.1M sulfuric acid on ice and left to rotate in the cold room for 2.5 h for histone extraction. 0.5 mL SP Fast Flow Sepharose beads (Cytiva) were washed with 5 mL loading buffer (300 mM NaCl, 50 mM Tris-HCl pH 8.0, 2 mM NaEDTA pH 8.0, 0.25 mM PMSF, 1 mM DTT), rotated 15 min in the cold room and centrifuged at 2000rpm for 5 min (4°C). This was repeated one more time.

After 2.5 h, sulfuric acid supernatant was centrifuged at 20,000 x g for 20 min to remove precipitate. The supernatant was collected again into a single tube and neutralised with 5 mL of 1M Tris-CL pH 8.0. Then topped up to 10 mL with more Tris. The supernatant was adjusted to loading buffer before mixing with the Sepharose beads and samples were left to rotate 1 h in the cold room to allow histones to bind. After being centrifuged at 2000 rpm for 5 min, the beads were washed with 5 mL wash buffer (400 mM NaCl, 50 mM Tris-HCl pH 8.0, 2 mM NaEDTA pH 8.0, 0.25 mM PMSF, 1 mM DTT). The samples were rotated in the cold room for 5 min before centrifuging and this was repeated a total of 3 times.

The histones were then eluted with 1.5 mL elution buffer (2000 mM NaCl, 50 mM Tris-HCl pH 8.0, 2 mM NaEDTA pH 8.0, 0.25 mM PMSF, 1 mM DTT) by rotating in the cold room for 5 min. The supernatant was collected in a separate tube after centrifuging at 2000 rpm for 5 min. The histones were precipitated in 4% perchloric acid rotating in the cold room overnight.

Samples were centrifuged at 18,500 x g for 1 h 15 min at 4°C and pellets were washed twice with 4% perchloric acid (first with 1 mL and then with 0.5 mL) followed by 0.5 mL 0.2% HCl in Acetone wash and centrifuged again at 18,500xg for 10 min. The last wash was done with 1 mL Acetone. Supernatant was aspirated carefully after centrifugation and tubes were left open for 5–10 min at RT for leftover acetone to evaporate.

Protein concentrations were measured by Bradford Assay. The pellets were resuspended in 0.25 mM PMSF in water. 10 μL of the sample was mixed with 200 μL Bradford Solution (Bio-Rad) and 790 μL water in a spectrophotometer cuvette and mixed by pipetting up and down.

1 μg of each sample was loaded and run on 18% gel (1 mm thick) for 1.5 h at 200V until the blue 6 kDa band of the SeeBlue Plus2 Pre-stained Protein Standard (Invitrogen) ladder reached the bottom of the gel. The gel was stained with Imperial Protein Stain (Coomassie dye R-250, Thermo Scientific). The gel images were taken on the Bio-Rad ChemiDoc MP.

Histone preparation for LC-MS, LC-MS fractionation and Skyline data analysis.

Performed as in.^28^

### Quantification and statistical analysis

Histone mass spectrometry data, analyzed in Skyline,^91^ was organized and imported into RStudio. All histone PTM plots were generated using R,^92^ particularly ggplot2.^94^ Kruskal-Wallis test followed by Conover-Iman test was used to determine whether there is a significant difference between the groups. The *p*-values for each comparison were adjusted with Benjamini–Hochberg procedure. The number of replicates (immunofluorescence experiments or independent histone preparations) is indicated in the figure legends. The bars are shown as mean ± standard deviation.

## Supplemental information

Document S1. Figures S1 and S2 and Data S2

Data S1. Mendeley links for raw datasets
