# Supplementary material for: Massive histone H3 phosphorylation without mitotic chromosome condensation
Source: iScience. 2026 Sep 22;29(10):117718. doi: 10.1016/j.isci.2026.117718 (PMC13675386; doi:10.1016/j.isci.2026.117718)
Supplement: Supplementary file 1 [file mmc1.pdf]

iScience, Volume *29*

Supplemental information

Massive histone H3 phosphorylation without mitotic chromosome condensation

Ipek Ustun, Marco Borsò, Natalia Y. Kochanova, Ignasi Forne, Caitlin Reid, Bram Prevo, Kumiko Samejima, Linfeng Xie, James R. Paulson, Axel Imhof, and William C. Earnshaw

A

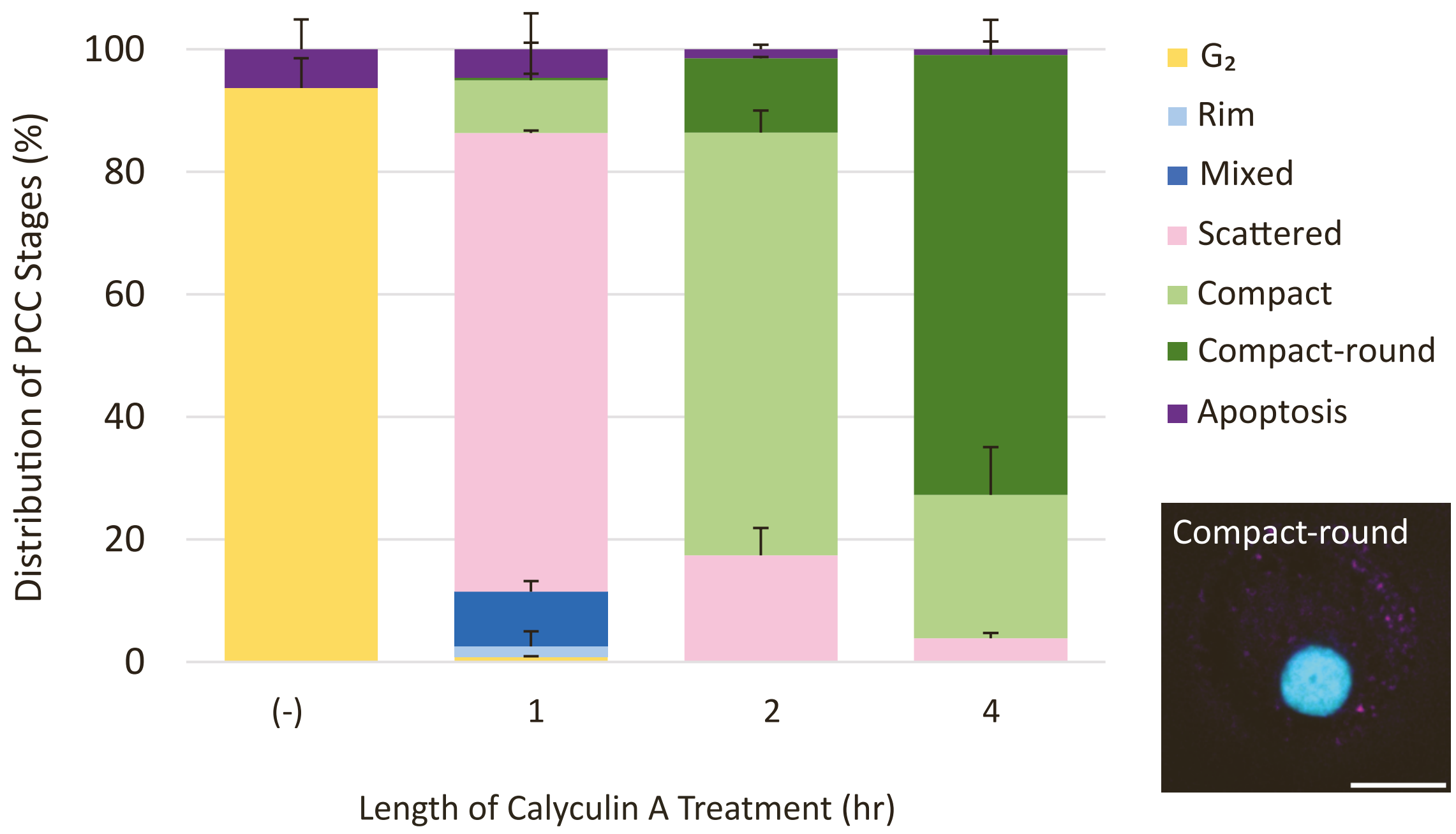

100
80
60
40
20
0
Distribution of PCC Stages (%)
(-)
1
2
4
Length of Calyculin A Treatment (hr)
G2
Rim
Mixed
Scattered
Compact
Compact-round
Apoptosis
Compact-round

B

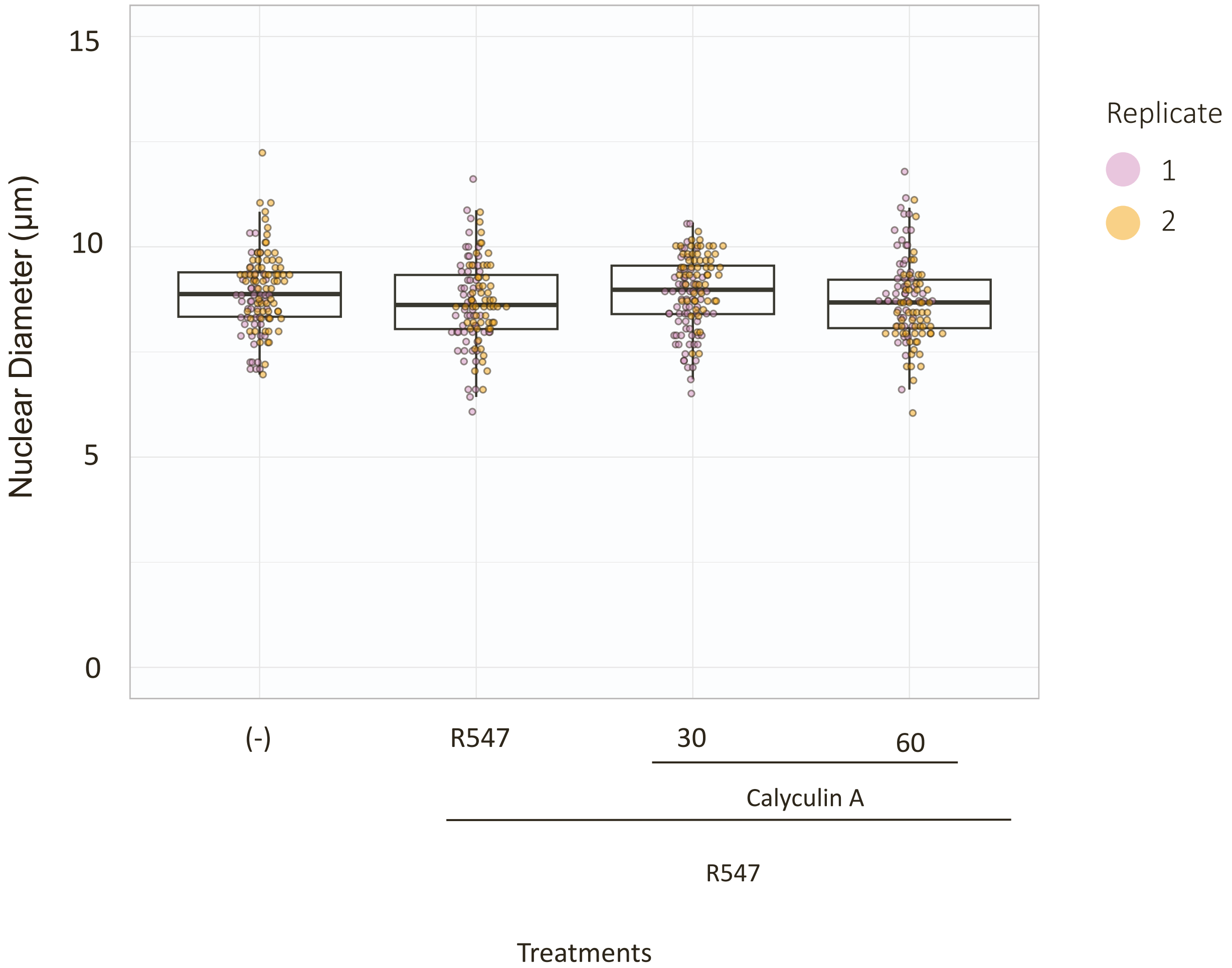

15
10
5
0
Nuclear Diameter (μm)
(-)
R547
30
60
Calyculin A
R547
Treatments
Replicate
1
2

Figure S1. Nuclei do not divide under calyculin A within 4 hrs. A) Distribution of PCC stages across different lengths of calyculin A treatment up to 4 hours. Negative (-) control contains G_2 arrested cells without calyculin A. The bars represent the mean ± standard deviation (N = 2). Deconvolved immunofluorescence image of what we called the compact-round phenotype is shown in right corner. DNA is in cyan and Lamin B1 is in magenta. Scale bar is 5 µm. B) Nuclear diameter of cells under calyculin A and/or R547 treatment. Nuclear diameters of 50-70 cells per treatment per replicate have been measured by drawing two lines across the nucleus stained with Lamin B1 at 90 degrees angle and taking the mean of the two measurements. Each final diameter has been plotted individually, separated by colour for replicates (N =2). Unlike others, images for these measurements were obtained with 60X objective.

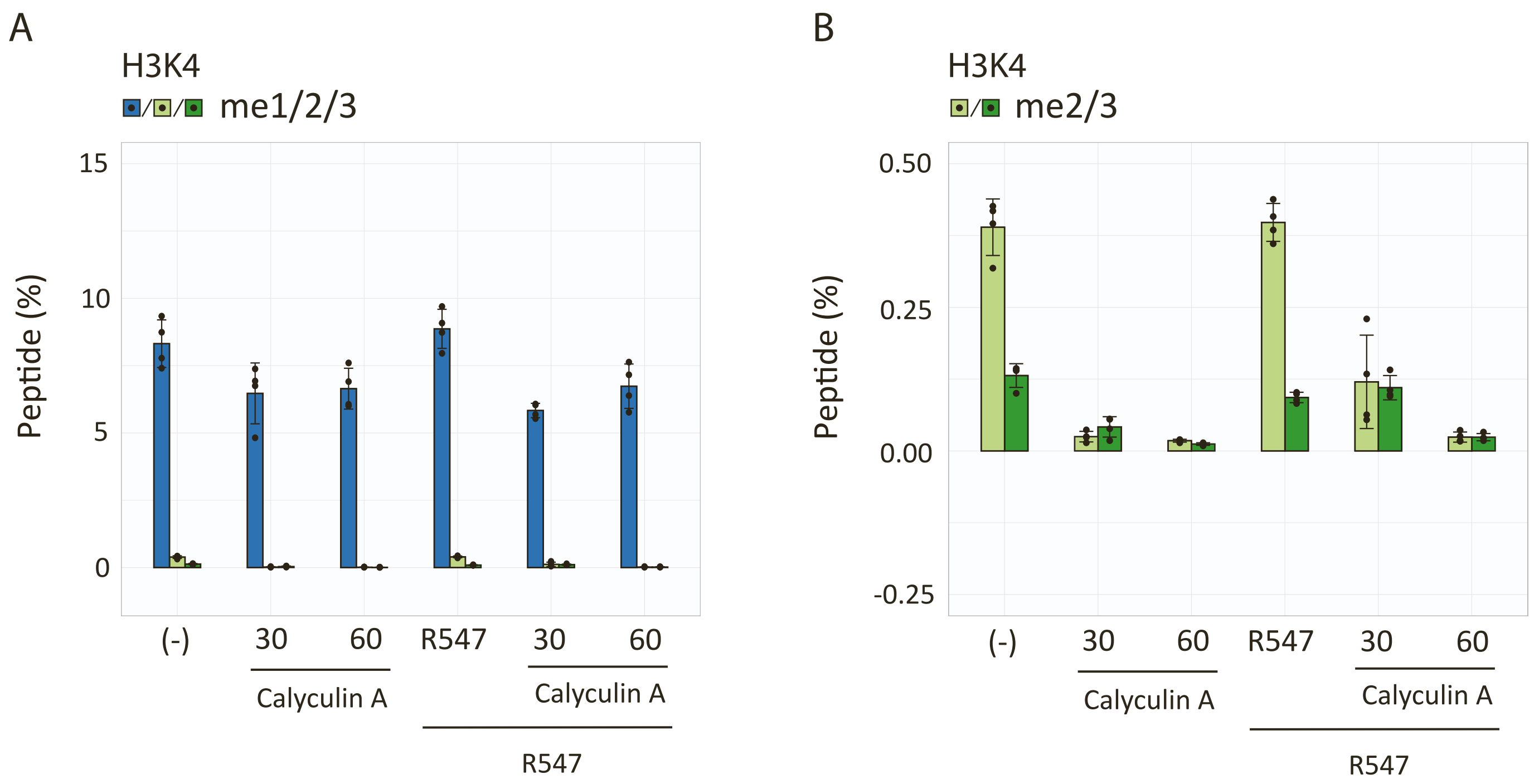
A
H3K4
me1/2/3
15
10
5
0
Peptide (%)
(-)
30
60
R547
30
60
Calyculin A
Calyculin A
R547
B
H3K4
me2/3
0.50
0.25
0.00
-0.25
Peptide (%)
(-)
30
60
R547
30
60
Calyculin A
Calyculin A
R547

Figure S2. Small scale changes in H3K4me2 and H3K4me3 under calyculin A. A) Mean peptide percentage of H3K4me1, me2 and me3 under calyculin A and/or R547, without me0 peptide percentage (N = 4). Error bars represent standard deviation. B) Same as in A but without H3K4me1 leaving H3K4me2 and me3 on a small scale (-0.25 – 0.5%) (N = 4).

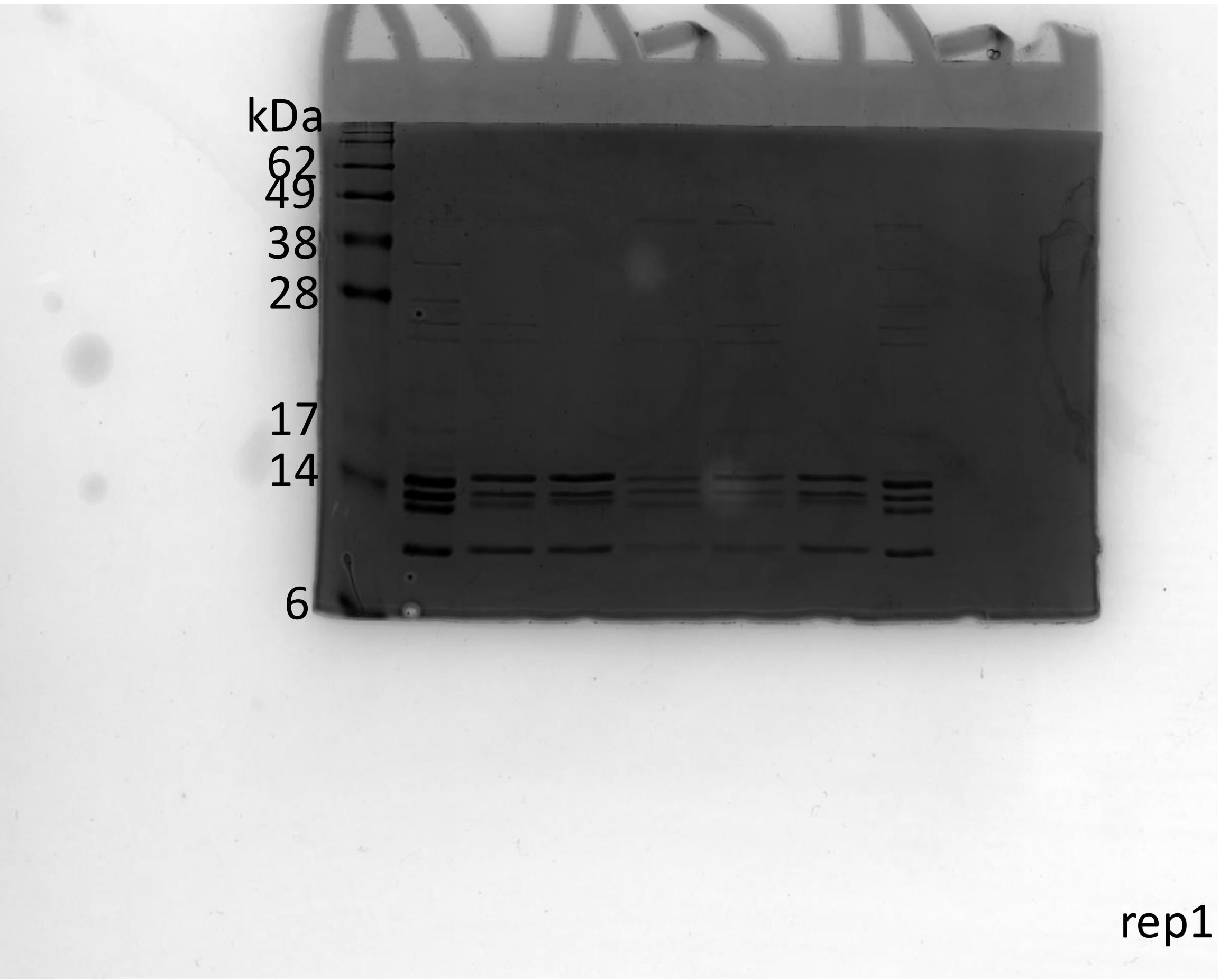
kDa
62
49
38
28
17
14
6
rep1

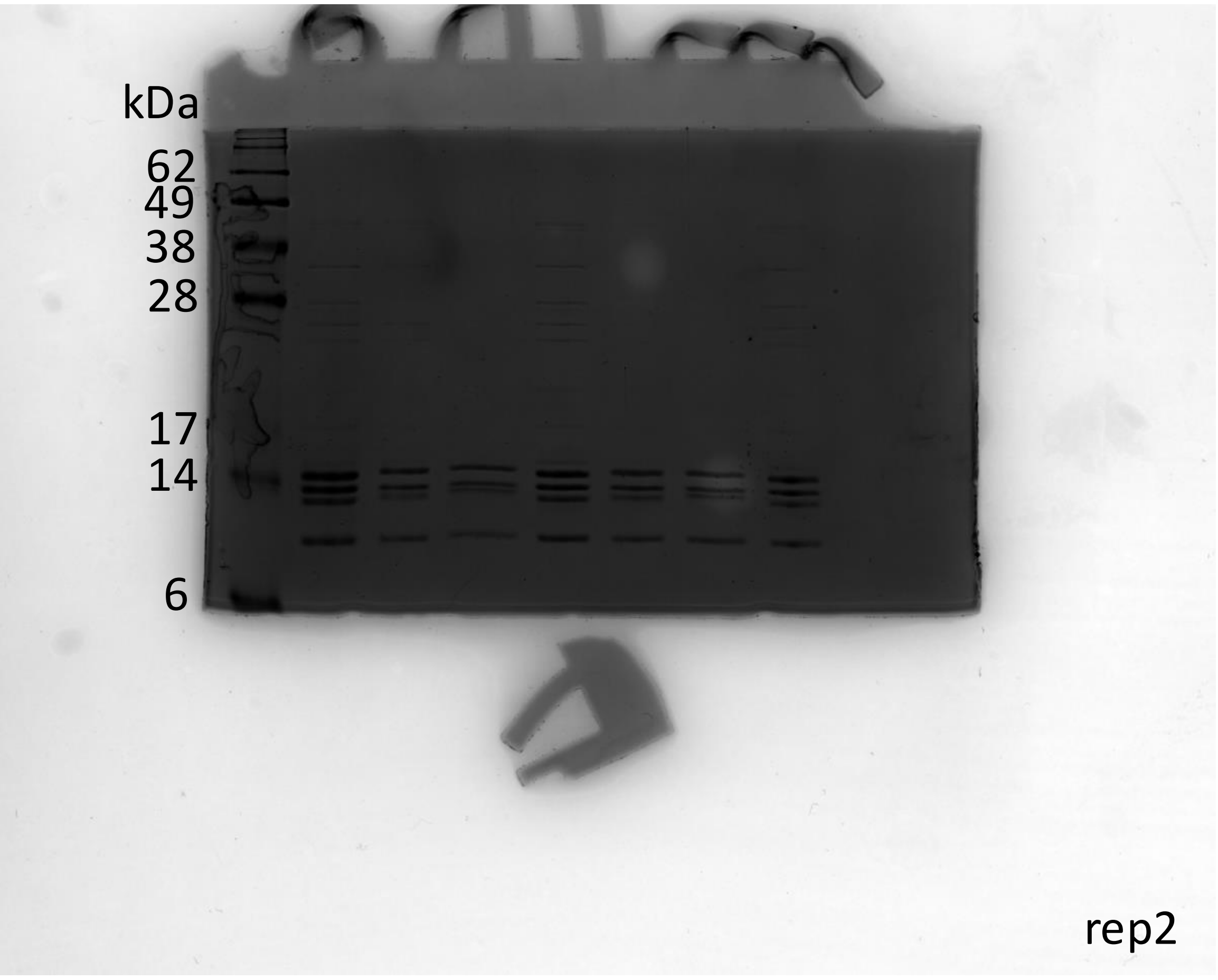
kDa
62
49
38
28
17
14
6
rep2

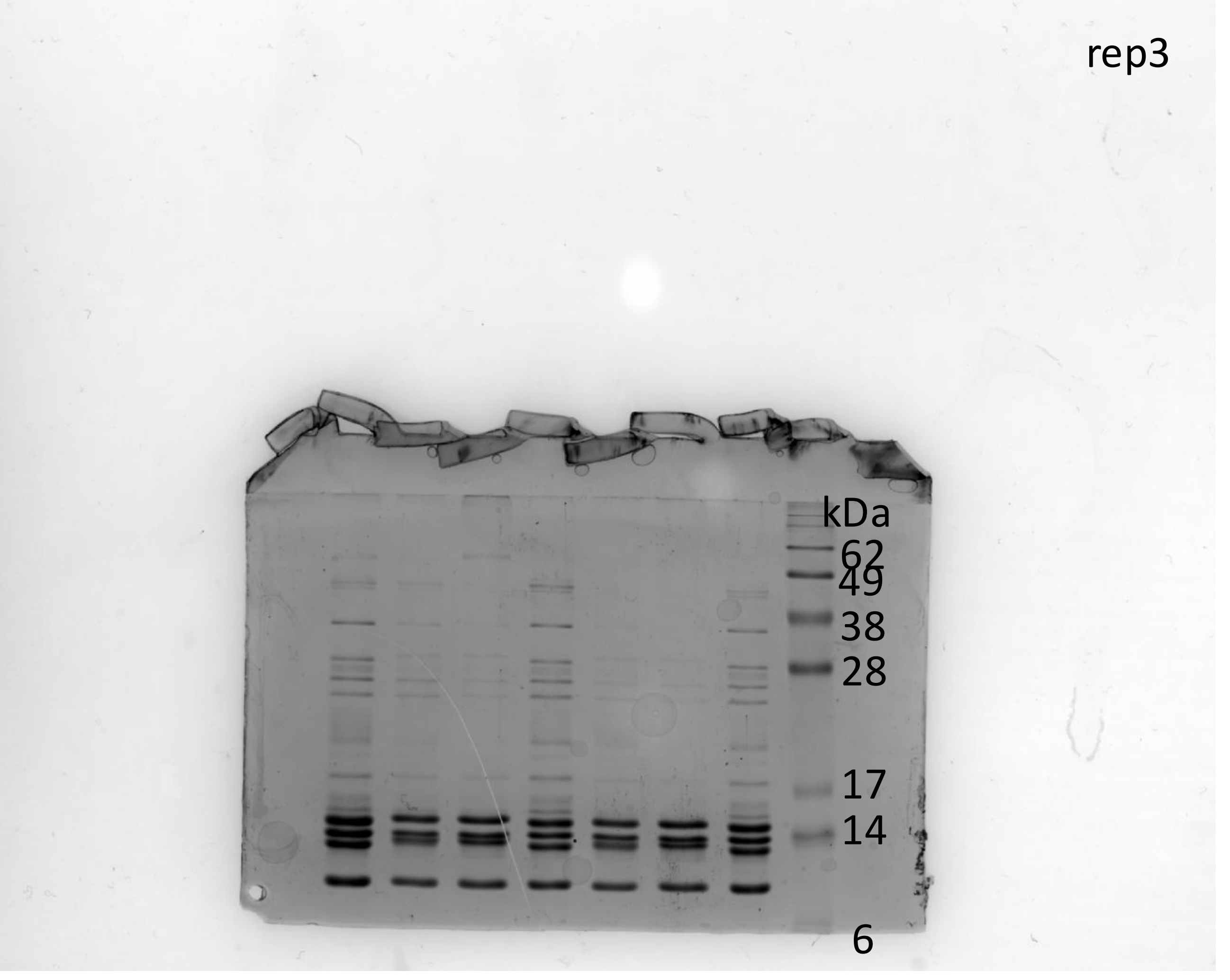
rep3
kDa
62
49
38
28
17
14
6

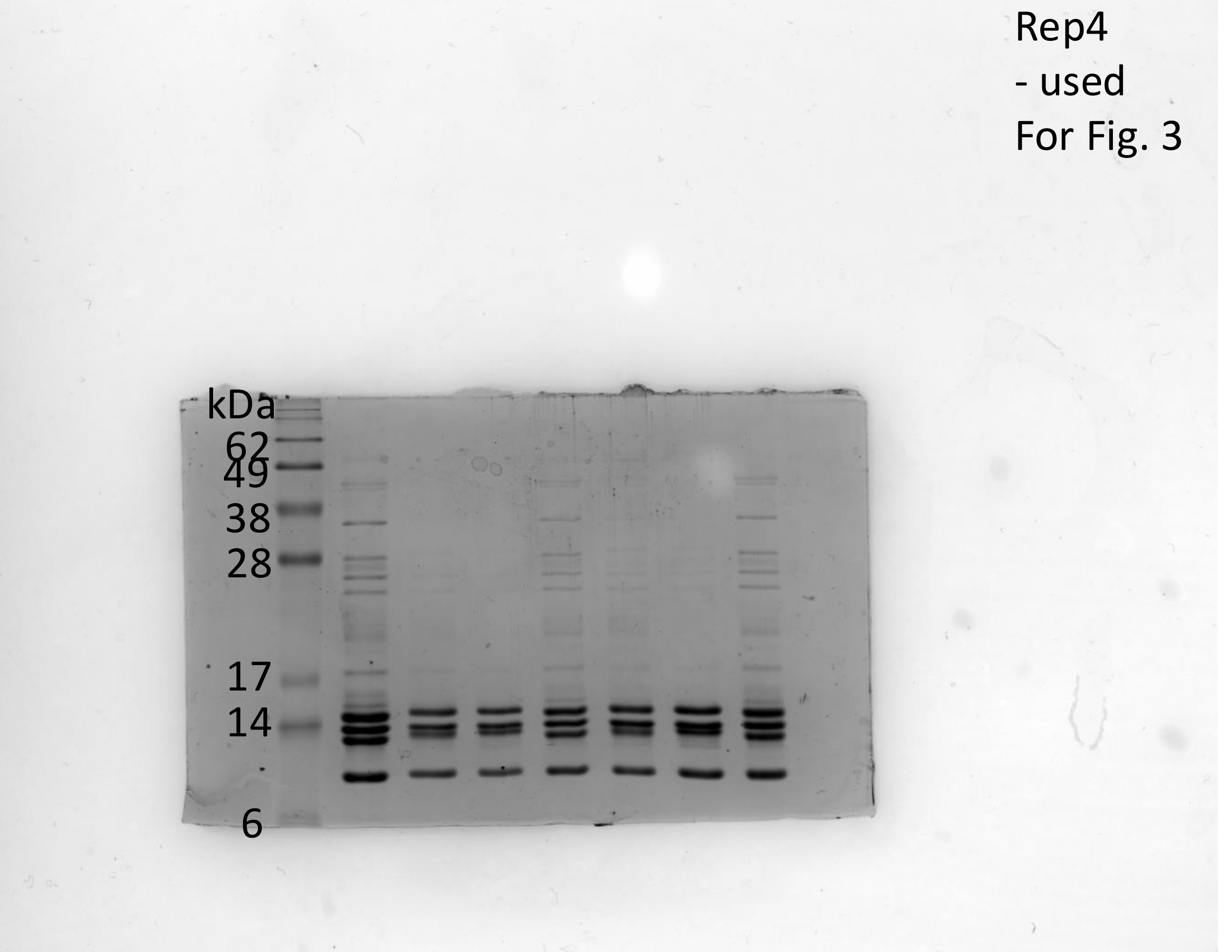
Rep4
- used
For Fig. 3
kDa
62
49
38
28
17
14
6

Data S2: Four western blot replicates of the experiment shown in Figure 3B
