# Supplementary material for: Massive histone H3 phosphorylation without mitotic chromosome condensation
Source: iScience. 2026 Sep 22;29(10):117718. doi: 10.1016/j.isci.2026.117718 (PMC13675386; doi:10.1016/j.isci.2026.117718)
Supplement: Supplementary file 2 [file mmc2.pdf]

Dataset name	Contents	Preview link
Massive histone H3 phosphorylation without mitotic chromosome condensation - I	Fig. 2 (R547/Palbo expt microscopy) - rep 1 part 1<br>Fig. 3 (histone preps microscopy) - reps 1 and 2	https://data.mendeley.com/preview/txfcb7wpps?a=a00caa2e-4650-4fc6-991c-4c3e1082c5d8
Massive histone H3 phosphorylation without mitotic chromosome condensation - II	Fig. 2 (R547/Palbo expt microscopy) - rep 1 part 2<br>Fig. 3 (histone preps microscopy) - reps 3 and 4 + processed images Fig. 3	https://data.mendeley.com/preview/hbkb3rmywr?a=82243cc1-0fb6-42b2-9470-e205285fe7af
Massive histone H3 phosphorylation without mitotic chromosome condensation - III	Fig. 2 (R547/Palbo expt microscopy) - rep 2 part 1<br>Fig. S1B (Nuclear Diam microscopy) - rep1	https://data.mendeley.com/preview/jyhy7vhkcj?a=ea328051-367d-469e-92b4-78d5e084ab62
Massive histone H3 phosphorylation without mitotic chromosome condensation - IV	Fig. 2 (R547/Palbo expt microscopy) - rep 2 part 2 + processed images Fig. 2<br>Fig. S1B (Nuclear Diam microscopy) - rep2 + raw data excel sheet	https://data.mendeley.com/preview/36ph8r7f7d?a=d8a3feec-de24-45cd-9ead-9f0e7666f77c

Massive histone H3 phosphorylation without mitotic chromosome condensation - V	Fig. 1 (G2-PCC short timecourse microscopy) - reps 1 (031123) and 2 (151123) + processed images Fig. 1<br>Fig. S1B (G2-PCC extended timecourse microscopy) - reps 1 (20230908) and 2 (20241113) + processed image Fig. S1B<br>Histone gel images - reps 1-4	https://data.mendeley.com/preview/3w758529g3?a=6d9fbc38-9f52-43ec-aa1e-8d88ec8ac51d
